# Silica gel-immobilized multidisciplinary materials applicable in stereoselective organocatalysis and HPLC separation†

**DOI:** 10.1039/c7ra12658a

**Published:** 2018-01-03

**Authors:** J. Tůma, M. Kohout

**Affiliations:** Department of Organic Chemistry, University of Chemistry and Technology Prague Technická 5, Prague 6 166 28 Czech Republic michal.kohout@vscht.cz

## Abstract

In this pilot study, we present novel bifunctional silica gel-immobilized materials applicable as heterogeneous organocatalysts and stationary phases in HPLC. The materials provided high stereoselectivity in both batch and continuous flow catalysis of a model Michael addition (cyclohexanone to (*E*)-β-nitrostyrene). In the batch reaction, the catalysts proved their sustainable catalytic activity over five consecutive recycling experiments. Under continuous flow reaction conditions, the catalytic activity was found to be superior to the batch reaction, and moreover, the same immobilized materials were utilized as stationary phases in HPLC showing very good chemoselective separation of model acidic analytes.

## Abstract 2

Novel multidisciplinary silica gel-based materials were successfully employed in highly stereoselective Michael addition and as stationary phases in HPLC separation.

## Graphical abstract

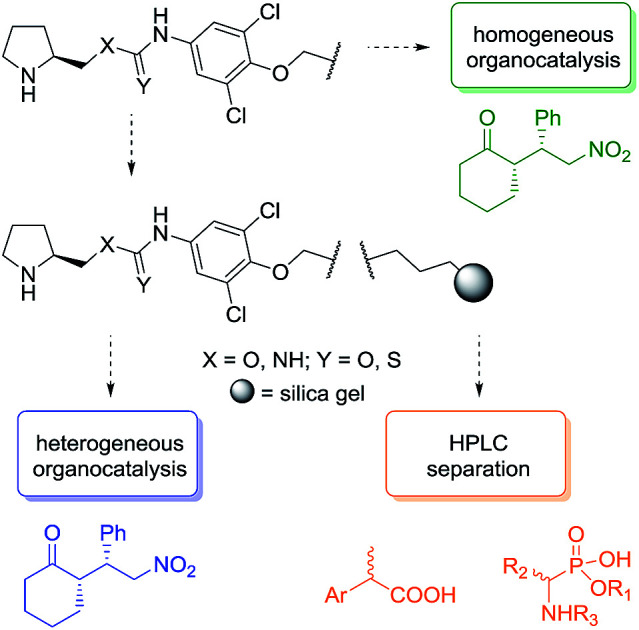

## Introduction

Organocatalysis stands amongst the major areas of contemporary stereoselective synthesis.^1–6^ Since its foundation on the verge of the new millennia,^7,8^ stereoselective organocatalysis has become widely exploited in countless organic reactions including Michael additions.^9–15^

In the past two decades, many organocatalysts have proven to be very effective in Michael additions of C-nucleophiles (aldehydes, ketones, malonates) to nitroolefins.^16–24^ To ensure high stereoselectivity of the reaction, bifunctional organocatalysts bearing secondary or tertiary amines together with a second activating moiety, such as urea, or thiourea, have also been developed.^25–27^

The bifunctional organocatalysts have demonstrated their high efficiency in both yield and stereoselectivity of the studied reactions, however they suffer from a rather troublesome recovery, which usually leads to a partial loss of the catalyst. The current approach to address this problem involves immobilization of the catalyst to a solid support.^28^ Several types of solid matrices, such as polystyrene,^29–35^ metal nanoparticles,^36,37^ metal–organic frameworks,^38^ ion-modified resins for non-covalent binding,^39–41^ or mesoporous silica^42–45^ have been introduced as solid supports for various types of organocatalysts.

Silica gel represents a well-defined and mechanically stable solid support for a large scale of organocatalytic species and ensures repeatable quantitative recycling of the heterogenized catalysts.^46^ Therefore, extensive studies of various chiral organocatalysts covalently bound to the silica gel surface were carried out, exploiting mostly l-proline, or *Cinchona* derivatives. Such materials were (amongst others) employed in asymmetric aldol reactions,^47–51^ Michael additions,^52–56^ three-component reactions,^57,58^ Diels–Alder reactions,^59–62^ or alkylations.^63^ In general, the utilized organocatalysts exhibited moderate to excellent conversions as well as stereoselectivity and good recyclability.^46^ Further studies were focused on non-covalently immobilized organocatalysts bound to ion liquid-modified silica gel surface.^64,65^

The sole immobilization of the catalyst, however, is not sufficient enough to meet the high demands of a potential large scale application. If the immobilized catalyst is used in a batch reaction system, it still requires a separation step (usually filtration) to be properly regenerated. On the other hand, if the catalyst is incorporated into a flow reactor, the regeneration requirements are easily achievable by a simple washing step. Several recent publications have been focused on this topic exploiting various types of solid supports,^66^ mostly polystyrene,^67–70^ or silica gel.^71–73^

Complementary to stereoselective synthesis, optically pure compounds can be achieved by chiral separation of racemates using chromatographic techniques.^74–77^ In such a case, a proper design of a suitable chiral stationary phase (CSP) bearing a chiral selector is essential in order to achieve good chromatographic resolution.

**Scheme 1:**
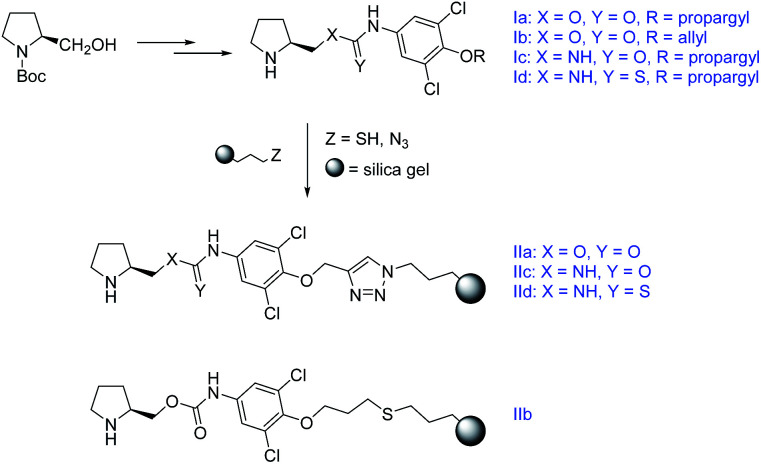
The synthetic pathway towards the immobilized compounds. For Experimental details see ESI.†

In this work we present a study of four chiral functionalized derivatives of carbonic acid (two carbamates, one urea, one thiourea) terminated by an alkynyl, or alkenyl chain, which enables efficient immobilization of the prepared compounds to a modified silica gel (Scheme 1). The prepared compounds were successfully employed as highly stereoselective homogeneous organocatalysts in model Michael addition of cyclohexanone to (*E*)-β-nitrostyrene (ee up to 95%, *syn* : *anti* up to 97 : 3). After immobilization to the silica gel solid support, the materials were exploited as heterogeneous catalysts in the same model reaction (ee up to 90%, *syn* : *anti* up to 95 : 5). The immobilized catalysts allowed for an easy regeneration with constant stereoselective performance and only partial decrease in reaction conversion. Moreover, the prepared materials were packed into stainless steel columns and tested in a continuous flow arrangement. Despite modest reaction conversions in this setup, higher stereoselectivity to the batch reaction experiments was obtained. The column-packed compounds were further utilized as stationary phases for separation of model acidic compounds (2-arylpropionic acids, *N*-protected 2-aminophosphonic acid mono-esters) using HPLC.

To our best knowledge, the presented materials are the first immobilized compounds applicable in highly stereoselective homogeneous and heterogeneous catalysis (both batch and flow setup) as well as in HPLC separation.

## Results and discussion

**Scheme 2:**
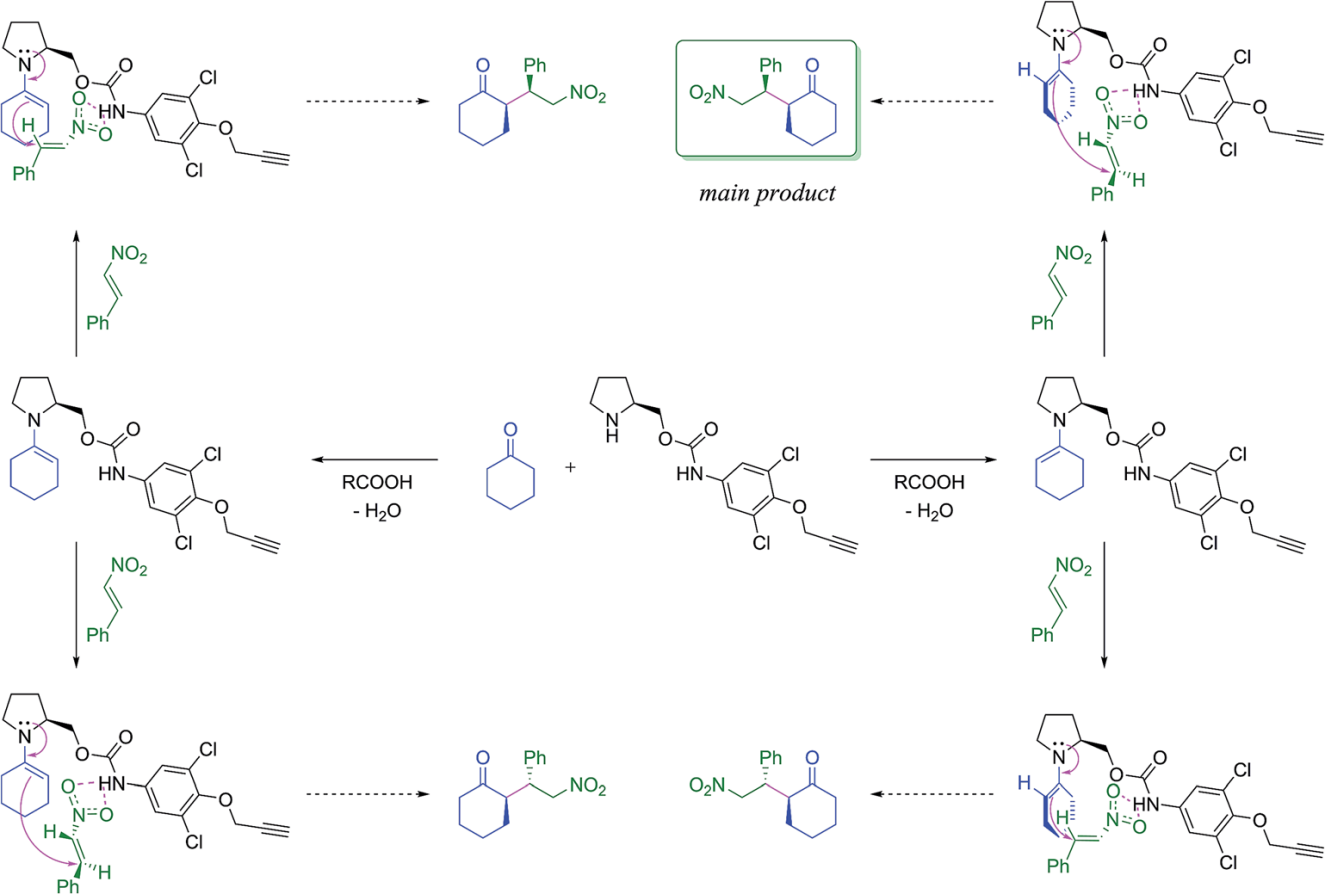
The proposed catalytic pathways towards the products of the studied Michael addition. For further details see ESI.†

First, the amino carbamate compound Ia was employed in a model Michael addition of cyclohexanone with (*E*)-β-nitrostyrene. The proposed reaction mechanisms towards all stereoisomers of the product is shown in Scheme 2. The reaction was carried out at room temperature and 0 °C in various solvents (Table 1). Butyric, or acetic acid was used as a co-catalyst mediating imine bond formation between the catalyst and cyclohexanone as well as supporting the hydrolysis of the corresponding iminium salt formed after the addition to nitrostyrene.^27^

**Table 1:** Model Michael addition in homogeneous environment catalyzed with Ia^a^

Entry	Catalyst	Solvent	Conversion^b^ (isol. yield^c^) [%]	ee^d^ [%]	*syn* : *anti*^b^
1	Ia	Hexane	73 (56)	84	92 : 8
2	Ia	Toluene	86 (70)	81	94 : 6
3	Ia	DCM	61 (48)	75	92 : 8
4	Ia	MeOH	30 (14)	51	82 : 18
5	Ia	Neat^e^^,^^f^	94 (87)	91	95 : 5
6	Ia	Neat^e^^,^^f^^,^^g^	74 (62)	93	95 : 5

a Reaction conditions: nitrostyrene 0.25 mmol, cyclohexanone 250 μl, Ia 0.05 mmol, butyric acid 0.0025 mmol, solvent 1 ml, *t* = 25 °C, reaction time = 48 h.

b Determined by ^1^H NMR.

c Purified by column chromatography (hexane : ethyl acetate = 5 : 1).

d Determined by chiral HPLC (column YMC Amylose C, mobile phase heptane : IPA = 9 : 1, flow rate 0.7 ml min^−1^).

e Additional 750 μl of cyclohexanone was used instead of the solvent.

f 0.035 mmol of AcOH used instead of butyric acid.

g Reaction carried out at *t* = 0 °C, reaction time = 144 h.

The amino carbamate compound Ia has proven to be a potent catalyst for the studied reaction. A highly impactful effect of the chosen solvent was observed. In general, the less polar solvent was used, the higher yield and stereoselectivity was achieved (Table 1, entries 1–4). The only exception was a slightly lower yield obtained in hexane (Table 1, entry 1) than in toluene (Table 1, entry 2) due to the lower solubility of the catalyst in hexane.

The best results, however, were found using neat cyclohexanone in the absence of solvent (Table 1, entries 5–6). At room temperature, the reaction proceeded almost quantitatively with 91% ee and high *syn*-stereoselectivity (95 : 5). Lowering the reaction temperature to 0 °C led to a slight improvement in stereoselectivity (ee = 93%), while minor decrease in the reaction conversion was observed (74%).

The other compounds from the series (Ib–d) were tested in the same model Michael addition (Table 2) using the previously obtained optimized conditions (Table 1, entry 5).

**Table 2:** Model Michael addition in homogeneous environment catalyzed with Ia–Id under the optimized conditions^a^

Entry	Catalyst	Reaction time [h]	Conversion^b^ (isol. yield^c^) [%]	ee^d^ [%]	*syn* : *anti*^b^
1	Ia	48	94 (87)	91	95 : 5
2	Ib	72	Quant. (91)	94	94 : 6
3	Ic	48	0	—	—
4	Id	5	Quant. (79)	84	95 : 5
5	Id (10 mol%)	48	Quant. (85)	92	97 : 3
6	Id (5 mol%)	120	50 (42)	93	95 : 5
7	Id (2 mol%)	120	7 (4)	93	96 : 4

a Reaction conditions: nitrostyrene 0.25 mmol, cyclohexanone 1 ml, catalyst 0.05 mmol, acetic acid 0.035 mmol, solvent 1 ml, *t* = 25 °C.

b Determined by ^1^H NMR.

c Purified by column chromatography (hexane : ethyl acetate = 5 : 1).

d Determined by chiral HPLC (column YMC amylose C, mobile phase heptane : IPA = 9 : 1, flow rate 0.7 ml min^−1^).

According to our expectations, the amino carbamate compound Ib provided comparable results to its direct analogue Ia (compare Table 2, entries 1 and 2). In the case of Ib, the reaction time was deliberately prolonged in order to reach quantitative conversion.

**Scheme 3:**
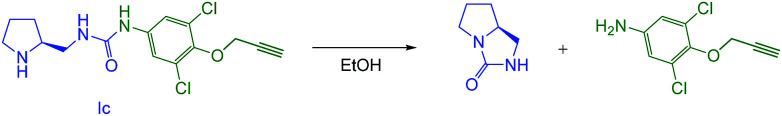
The decomposition of amino urea Ic.

Despite the fact that most of the current bifunctional organocatalysts employ a urea or thiourea moiety, in our case the amino urea-type compound Ic provided only traces of the desired product (Table 2, entry 3). The low to none activity of the catalyst can be ascribed to its low stability. Surprisingly, in contrast with its amino carbamate analogues Ia,b, it is prone to spontaneous decomposition even at ambient temperature. This decomposition is initiated by an intramolecular nucleophilic attack of the secondary amino group of the catalyst to the urea moiety followed by the detachment of corresponding aromatic amine. This leads to the quick deactivation of the catalyst. We have confirmed this undesired reaction mechanism by isolating the decomposition products after treatment of Ic with ethanol at reflux (Scheme 3; for experimental details see ESI†).

The amino thiourea compound Id exhibited highly increased catalytic activity in the model reaction. A full conversion was obtained within only 5 h (Table 2, entry 4). Therefore, following optimization regarding the amount of the catalyst was carried out (Table 2, entries 5–7). 10 mol% of the catalyst Id provided quantitative conversion within acceptable time span (48 h) and excellent stereoselectivity (Table 2, entry 5). Further decrease of the catalyst amount led to protracted reaction time and a conversion drop without any significant effect on the reaction stereoselectivity (Table 2, entries 6, 7).

The catalytic activity of the amino thiourea compound Id was further studied in the same solvents as in the case of Ia (Table 3). Based on the previous experiments, only 10 mol% of the catalyst was used in the reactions.

**Table 3:** Model Michael addition in homogeneous environment catalyzed with Id^a^

Entry	Solvent	Reaction time [h]	Conversion^b^ (isol. yield^c^) [%]	ee^d^ [%]	*syn* : *anti*^b^
1	Hexane	72	61 (32)	95	94 : 6
2	Toluene	48	Quant. (92)	89	91 : 9
3	DCM	72	77 (52)	80	90 : 10
4	MeOH	72	0	—	—

a Reaction conditions: nitrostyrene 0.25 mmol, cyclohexanone 250 μl, Id 0.025 mmol, acetic acid 0.035 mmol, solvent 1 ml, *t* = 25 °C.

b Determined by ^1^H NMR.

c Purified by column chromatography (hexane : ethyl acetate = 5 : 1).

d Determined by chiral HPLC (column YMC Amylose C, mobile phase heptane : IPA = 9 : 1, flow rate 0.7 ml min^−1^).

The obtained results have confirmed the previously observed trend of solvent influence. The less polar solvent is used, the higher stereoselectivity is obtained (Table 3). While no product was formed in methanol, the stereoselectivity found in hexane (Table 3, entry 1) even equaled, if not exceeded, the original experiment in neat cyclohexanone (compare to Table 2, entry 5). The use of hexane, thus, overcomes the necessity of the neat reaction conditions, which are rather inconvenient in terms of a potential practical application.

**Scheme 4:**
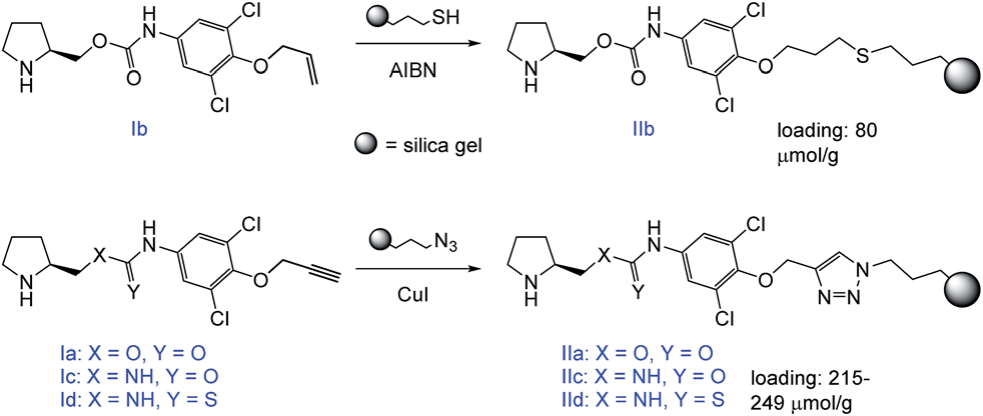
Immobilization of the target compounds.

The prepared compounds were further immobilized to a modified silica gel. Either a radical-initiated addition of 3-mercaptopropyl-modified silica gel to the terminal double bond (Ib), or a click-reaction of 3-azidopropyl-modified silica gel to the terminal triple bond (Ia,c,d) was used (Scheme 4).

The immobilized compound IIb was obtained in a total loading of 80 μmol g^−1^, which corresponds to a 30% reaction conversion. On the other hand, the materials IIa,c,d were yielded in the total coverage up to 249 μmol g^−1^, which corresponds to an almost quantitative immobilization.

The obtained heterogeneous catalysts IIa–d were tested in the same model Michael addition of cyclohexanone to (*E*)-β-nitrostyrene under the optimized reaction conditions as for Ia–d (Table 4).

**Table 4:** Model Michael addition in heterogeneous environment catalyzed with IIa–d^a^

Entry	Catalyst	Reaction time [h]	Conversion^b^ (isol. yield^c^) [%]	ee^d^ [%]	*syn* : *anti*^b^
1	IIa	72	51 (36)	78	95 : 5
2	IIb	72	49 (18)	90	90 : 10
3	IIc	72	10 (7)	58	83 : 17
4	IId	72	25 (10)	86	94 : 6

a Reaction conditions: nitrostyrene 0.25 mmol, cyclohexanone 1 ml, catalyst 0.05 mmol, acetic acid 0.035 mmol, *t* = 25 °C, reaction time = 72 h.

b Determined by ^1^H NMR.

c Purified by column chromatography (hexane : ethyl acetate = 5 : 1).

d Determined by chiral HPLC (column YMC Amylose C, mobile phase heptane : IPA = 9 : 1, flow rate 0.7 ml min^−1^).

Undoubtedly, in all cases the overall reaction conversion has decreased in comparison to the experiments in homogeneous environment. Surprisingly, the two amino carbamate catalysts (IIa,b) yielded almost the same conversion (Table 4, entries 1, 2), despite having significantly different loading of the organocatalyst (see Scheme 4). On the other hand, the amino thiourea catalyst IId, which was, based on the homogeneous experiments, expected to be the most active, delivered only 25% of the product (Table 4, entry 4). Based on these unexpected results, it can be concluded that the kinetics of the reaction in heterogeneous environment is not driven by the chemical reaction itself. The rate-limiting step is most probably denoted by the diffusion of either the reagents into the silica gel pores (where the catalyst molecules are bound), or the products from the pores into the solvent. Nevertheless, it is worth pointing out that the prepared immobilized compounds IIa,b,d have proven to be efficient stereoselective heterogeneous catalysts of the studied reaction (ee = 78–90%, *syn* : *anti* = 90 : 10–95 : 5).

The amino urea compound IIc delivered a very poor yield of the product with only moderate stereoselectivity (Table 4, entry 3). It is highly probable that the immobilized catalyst suffers from the same instability as its homogeneous precursor Ic (see Scheme 3). Therefore its activity is limited accordingly.

The prepared heterogeneous catalysts IIa,d were further tested in the same set of solvents as their homogeneous analogues (Table 5). For both catalysts, a similar trend of the solvent influence to the experiments in homogeneous environment was observed, *i.e.* the less polar solvent was used, the higher stereoselectivity was obtained (ee up to 82%, *syn* : *anti* up to 94 : 6).

**Table 5:** Model Michael addition in heterogeneous environment catalyzed with IIa and IId^a^

Entry	Catalyst	Solvent	Conversion^b^ (isol. yield^c^) [%]	ee^d^ [%]	*syn* : *anti*^b^
1	IIa	Hexane	44 (23)	82	92 : 8
2	IIa	Toluene	51 (38)	78	94 : 6
3	IIa	DCM	39 (16)	70	92 : 8
4	IIa	MeOH	16 (10)	46	82 : 18
5	IId	Hexane	37 (21)	80	94 : 6
6	IId	Toluene	23 (15)	78	94 : 6
7	IId	DCM	17 (10)	74	92 : 8
8	IId	MeOH	7 (3)	53	86 : 14

a Reaction conditions: nitrostyrene 0.25 mmol, cyclohexanone 250 μl, catalyst 0.05 mmol, acetic acid 0.035 mmol, solvent 1 ml, *t* = 25 °C, reaction time = 72 h.

b Determined by ^1^H NMR.

c Purified by column chromatography (hexane : ethyl acetate = 5 : 1).

d Determined by chiral HPLC (column YMC Amylose C, mobile phase heptane : IPA = 9 : 1, flow rate 0.7 ml min^−1^).

In the case of the amino thiourea material IId, the reaction in hexane notably surpassed the reaction conversion found in neat cyclohexanone (compare Table 4, entry 4 and Table 5, entry 5). Therefore, the reaction conditions in hexane were considered as the optimized conditions, despite minor decrease of the reaction stereoselectivity in comparison to the experiment in neat cyclohexanone. As mentioned above, solvent conditions are generally more convenient than the neat reaction conditions, since they allow for cheaper potential practical applications.

The achieved results are comparable to values found for a silica-supported pyrrolidine-triazole catalyst (ee up to 91%, *syn* : *anti* up to 94 : 6) under neat conditions.^52^ The studied organocatalysts slightly underperformed in comparison to an imidazolium–pyrrolidine silica-supported catalyst, for which very high values of enantio- and diastereoselectivity (ee up to 99%, *syn* : *anti* up to 99 : 1) together with yields up to 96% were reported.^53^ On the other hand, our results for the addition of cyclohexanone to (*E*)-β-nitrostyrene are much better than those achieved with a heterogeneous pyrrolidine-tetrazole organocatalyst (ee up to 55%, *syn* : *anti* up to 15 : 1), which was however primarily developed for continuous flow Michael addition of cyclohexanone to 4-nitrobenzaldehyde.^72^

The performance of the immobilized heterogeneous catalysts IIa and IId was subsequently studied in a series of recycling experiments. Five consecutive reactions under the optimized conditions (Table 4, entry 1 and Table 5, entry 5) with the same batch of the catalyst were carried out. After each reaction, the immobilized catalyst was filtered off the reaction mixture, washed and used in the next cycle (Table 6).

**Table 6:** Model Michael addition in heterogeneous environment catalyzed with IIa and IId^a^

Entry	Catalyst	Solvent	Conversion^b^ (isol. yield^c^) [%]	ee^d^ [%]	*syn* : *anti*^b^
1	IIa	neat^e^	51 (36)	78	95 : 5
2	IIa	Neat^e^	34 (17)	72	94 : 6
3	IIa	Neat^e^	37 (20)	70	96 : 4
4	IIa	Neat^e^	32 (18)	69	96 : 4
5	IIa	Neat^e^	35 (21)	69	93 : 7
6	IId	Hexane	37 (21)	80	94 : 6
7	IId	Hexane	21 (13)	80	95 : 5
8	IId	Hexane	20 (11)	89	92 : 8
9	IId	Hexane	22 (13)	81	94 : 6
10	IId	Hexane	19 (12)	79	93 : 7

a Reaction conditions: nitrostyrene 0.25 mmol, cyclohexanone 250 μl, catalyst 0.05 mmol, acetic acid 0.035 mmol, solvent 1 ml, *t* = 25 °C, reaction time = 72 h.

b Determined by ^1^H NMR.

c Purified by column chromatography (hexane : ethyl acetate = 5 : 1).

d Determined by chiral HPLC (column YMC Amylose C, mobile phase heptane : IPA = 9 : 1, flow rate 0.7 ml min^−1^).

e Additional 750 μl of cyclohexanone was used instead of the solvent.

Throughout the set of the experiments, both the immobilized materials IIa and IId proved their permanent catalytic activity. In the case of IIa, only a slight decrease of ee (7–9%) after the first reaction cycle was observed (Table 6, entries 1–5), whereas the catalyst IId yielded the same stereoselectivity through the whole reaction span (Table 6, entries 6–10).

In the consequent reactions, a minor drop of conversion was found after the first cycle, however the average value remained constant in further cycles (Table 6, entries 2–5 and 7–10). The decrease in the reaction conversion might be a consequence of a less effective re-activation of the catalyst by simple washing after each reaction cycle.

**Fig. 1:**
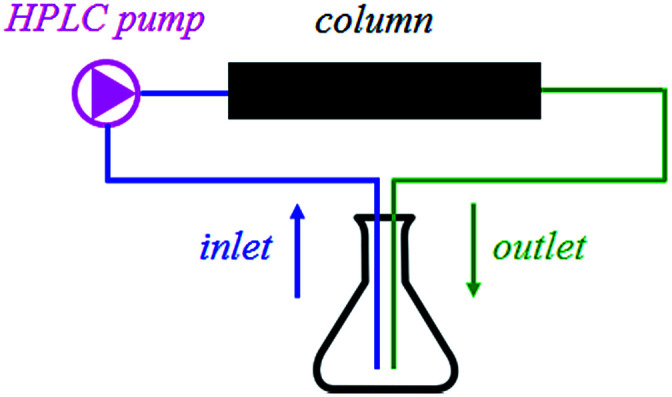
The experimental setup for continuous flow reaction.

Furthermore, the compounds IIa and IId were packed in-house into stainless steel columns (150 × 4 mm) and incorporated into a continuous flow system. As shown in Fig. 1, the system contains only one reaction mixture reservoir that works as both the inlet and outlet. Therefore, in terms of the reaction kinetics, the system does not run a flow reaction, but rather a batch reaction, which takes place inside the column. Nevertheless, the main advantage of the continuous flow system – no need for the catalyst separation and simple regeneration by washing – remains intact. The results are shown in Table 7. After the reaction, the columns were washed with 1% solution of acetic acid in methanol to obtain the fully regenerated catalyst for the next reaction cycle.

**Table 7:** Model Michael addition: continuous flow reaction catalyzed with IIa and IId

Entry	Catalyst	Solvent	Conversion^c^ (isol. yield^d^) [%]	ee^e^ [%]	*syn* : *anti*^c^
1^a^^f^	IIa	Neat	32 (24)	86	93 : 7
2^b^^f^	IId	Hexane	7 (5)	80	94 : 6

a Reaction conditions: nitrostyrene 5.0 mmol, cyclohexanone 100 ml, acetic acid 50.0 mmol, flow 0.7 ml min^−1^, *t* = 25 °C, reaction time = 72 h.

b Reaction conditions: nitrostyrene 5.0 mmol, cyclohexanone 100 ml, acetic acid 50.0 mmol, flow 0.7 ml min^−1^, *t* = 25 °C, reaction time = 72 h.

c Determined by ^1^H NMR.

d Purified by column chromatography (hexane : ethyl acetate = 5 : 1).

e Determined by chiral HPLC (column YMC Amylose C, mobile phase heptane : IPA = 9 : 1, flow rate 0.7 ml min^−1^).

f Residence time = 42 min.

In the case of IIa, the continuous flow reaction has delivered an equal conversion as the batch reaction using the same catalyst after the first reaction cycle (Table 6, entries 2–5). On the other hand, enantioselectivity has exceeded the original batch experiments yielding ee = 86%, while keeping the diastereoselectivity at a very high ratio of *syn* : *anti* = 93 : 7.

The compound IId yielded only 7% of the desired product. However, equally high stereoselectivity as in the batch reactions was obtained (ee = 80%, *syn* : *anti* = 94 : 6).

The overall low yields of the studied continuous flow setup could be explained by short residence time of the substrates inside the catalytic columns, which leads to their limited interaction with the catalyst. This issue as well as other catalytic properties can be addressed by an easy tunability of the catalytic system (varying the particle size and porosity of the solid support, extending the spacer length of the catalyst, *etc.*).

More efficient and already verified supports such as organic polymers^29–35^ or nanoparticles^36,37^ have, however, not been considered in this study. The obvious reason is that we aimed for simultaneous use of the new materials as continuous flow organocatalysts and stationary phases for HPLC. Therefore, the silica gel solid support for the materials IIa–d was selected.

The basic nature of the studied materials predetermines them for efficient interaction with acidic analytes in the sense of ion-exchange process. Under weakly acidic mobile phase conditions, the basic selector is protonated, while the analytes (organic acids) are fully or reversibly deprotonated. The electrostatic attraction between the charged functional groups is primarily responsible for retention of analytes, while additional interactions (π–π, hydrogen bonds, steric interactions) may facilitate chiral recognition of enantiomers.^78,79^

**Fig. 2:**
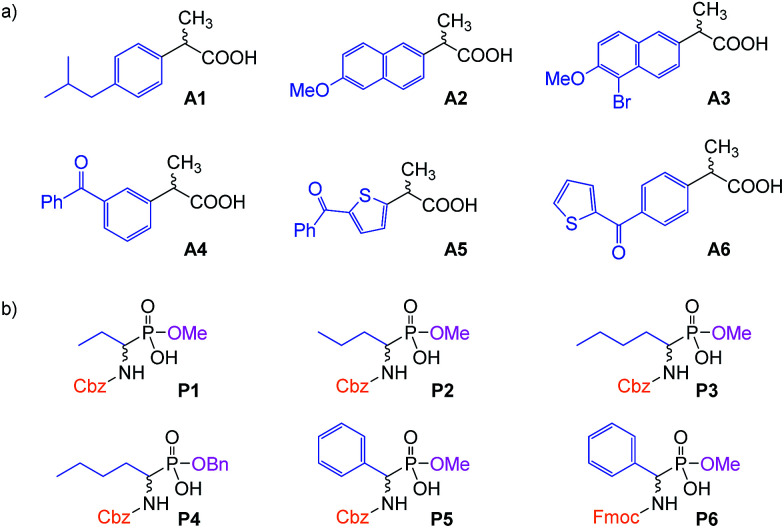
The studied analytes: (a) 2-arylpropionic acids; (b) *N*-protected 2-aminophosphonic acid mono-esters.

To test the properties of the prepared materials IIa–d as CSPs for the separation of acidic analytes, two classes of compounds were chosen (Fig. 2). Analytes A1–A6 (2-arylpropionic acids) belong among non-steroidal anti-inflammatory drugs and thus their purity (and enantiomeric purity) is often the target of quality control departments of pharmaceutical industries as well as research laboratories. For analytes P1–P6 (*N*-protected 2-aminophosphonic acid mono-esters), we have already reported efficient enantioseparation on analytical scale using quinine- and quinidine-based AX CSPs.^80^

**Fig. 3:**
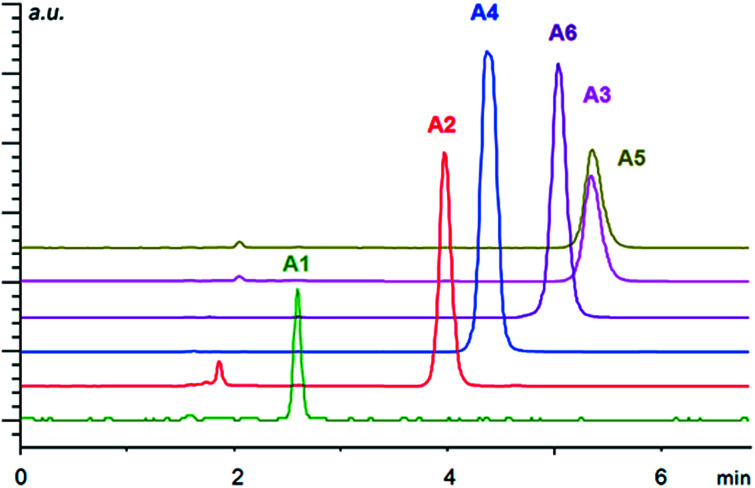
Separation of arylpropionic acids A1–A6 in ion exchange mode under HPLC conditions using the column-packed material IIa. Conditions: mobile phase, methanol/acetic acid/ammonium acetate (99/1/0.5, v/v/w%); flow rate of 1 ml min^−1^; temperature 20 °C. The injection volume was 5 μl with the sample concentration of 1 mg ml^−1^. The detection wavelength was set to 230 nm.

**Fig. 4:**
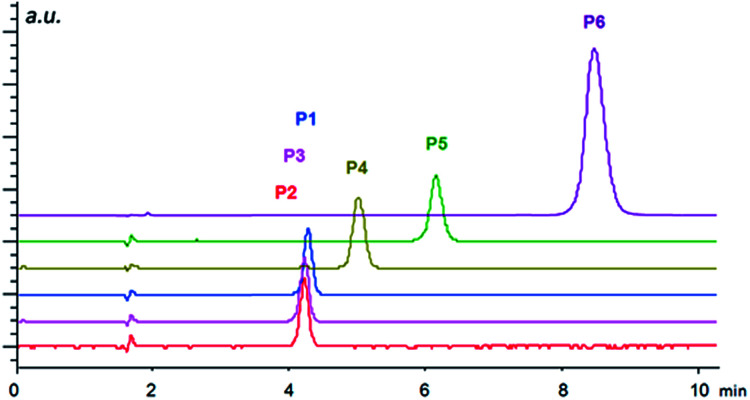
Separation of *N*-protected aminophosphonic acid mono-esters P1–P6 in ion exchange mode under HPLC conditions using the column-packed material IIa. Conditions: mobile phase, methanol/acetic acid/ammonium acetate (99/1/0.5, v/v/w%); flow rate of 1 ml min^−1^; temperature 20 °C. The injection volume was 5 μl with the sample concentration of 1 mg ml^−1^. The detection wavelength was set to 230 nm.

Although the studied immobilized compounds IIa–d did not afford enantioseparation of the individual analytes, they showed very good chemoselectivity allowing to separate a mixture of analytes of each class. Selected examples are shown in Fig. 3 and 4 (for other figures see ESI†). For the separation of various mixtures, reversed phase (RP) chromatography is commonly used. This technique takes the advantage of lipophilic stationary phases (usually silica modified with long alkyl chains) operated in a polar mobile phase. Separation is based on hydrophobic interactions of analytes with the stationary phase. RP chromatography has very broad application range,^81^ however, highly polar and ionic analytes may be badly resolved or not retained at all.^82^ Therefore the development of so-called mixed-mode stationary phases that offer combination of different interaction modes is of high interest nowadays.^83,84^ The presented materials can be considered as RP-ion-exchange systems.

Overall, the arylpropionic acids were eluted slightly faster than *N*-protected aminophosphonic acid mono-esters, which is in agreement with lower acidity (weaker electrostatic interaction) of arylpropionic acids in comparison with the aminophosphonic acid mono-ester derivatives. The lowest retention was found for ibuprofen (A1) with a flexible alkyl chain followed by naproxen (A2) (Fig. 3). On the other hand, the second highest retention time was observed for bromo-naproxen. It seems that the steric demand of the substituent connected to the central aromatic ring of the substance significantly influences its interaction with the selector.

The importance of the size of substituents is well documented by the separation of *N*-protected aminophosphonic acid mono-esters (Fig. 4). While mono-methyl esters P1–P3 based on aliphatic aminophosphonic acids possessing a benzyloxycarbonyl protecting group showed only minor differences in retention times, the introduction of bulkier ester group already led to considerable shift in retention time. Additional elongation of retention time was observed for phenylphosphonic mono-ester P5 and a dramatic shift towards higher retention was found in case of Fmoc-protected derivative P6. These results indicate that the primary electrostatic interaction is further supported by π–π interactions, which leads to higher retention. This enhanced interaction is, however, not sufficient to facilitate chiral recognition of the analytes, thus, both enantiomers still co-elute in one peak. This may be caused by the overall high flexibility of the immobilized compounds, which is important for the catalytic function, but does not provide a pre-organized binding site necessary for chiral recognition.

The only stationary phase that underperformed in the chemoselective separation of the analytes was the amino carbamate compound IIb. This material was prepared using a different immobilization technique (see Scheme 4), which yielded only low selector loading (80 μmol g^−1^). Thus, the stationary phase is not robust enough to effectively retain the analytes and therefore no separation is achieved (for chromatograms see ESI†).

## Conclusions

In conclusion, a series of silica gel-based materials bearing chiral bifunctional carbonic acid derivatives was designed and synthesized. The prepared compounds were successfully implemented as homogeneous as well as heterogeneous organocatalysts in a model Michael addition (cyclohexanone to (*E*)-β-nitrostyrene). In both cases, high stereoselectivity was obtained. The stereoselective activity of two of the immobilized materials has proven to remain stable over the span of five consecutive reaction cycles. Moreover, the prepared compounds were packed in-house into steel columns and tested in a continuous flow arrangement. Despite low reaction conversions, equal or higher stereoselectivity was found in comparison to the batch reaction experiments.

The prepared materials were further employed as stationary phases for the separation of model acidic analytes (2-arylpropionic acids, *N*-protected 2-aminophosphonic acid mono-esters). Very good chemoselective separation of both sets of analytes was achieved.

The presented silica gel-based materials are the first compounds reported that allow for both the stereoselective heterogeneous organocatalysis (batch and continuous flow setup) and separation in HPLC. The combined properties of highly potent organocatalysts for stereoselective Michael addition and stationary phases that allow separation (and potentially enantioseparation) of various analytes, open new door in the design and development of such type of multidisciplinary materials.

## Conflicts of interest

There are no conflicts to declare.

## Supplementary Material

RA-008-C7RA12658A-s001
