# Supplementary material for: Silica gel-immobilized multidisciplinary materials applicable in stereoselective organocatalysis and HPLC separation
Source: RSC Adv. 2018 Jan 3;8(3):1174–81. doi: 10.1039/c7ra12658a (PMC9076945; doi:10.1039/c7ra12658a)
Supplement: RA-008-C7RA12658A-s001 [file RA-008-C7RA12658A-s001.pdf]

Electronic Supplementary Material (ESI) for RSC Advances.
This journal is © The Royal Society of Chemistry 2017

Silica gel immobilized multidisciplinary materials applicable in stereoselective organocatalysis and HPLC separation

Jiří Tůma, Michal Kohout

Department of Organic Chemistry, University of Chemistry and Technology Prague, Technická 5, 166 28 Prague 6, Czech Republic

Electronic Supplementary Information

This ESI file contains details on the synthesis of the presented organocatalysts and analytical data for all intermediates and final products. The performance of the heterogeneous organocatalysts **IIb,c,d** as stationary phases for high-performance liquid chromatography (HPLC) is also reported.

Table of contents

Synthesis 1
Decomposition of Ic and IIc 5
Mechanism of the studied Michael addition 6
Experimental 8
HPLC separation using stationary phases IIb-d 24
NMR spectra 27
References 39

Synthesis

First, two key precursors **2** and **4** were synthesised (**Scheme ESI-1**). *N*-protected prolinol (**2**) was prepared via known methods, starting with $LiAlH_4$ reduction of *N*-Boc-*L*-proline (**1**) preceeded by methylation of the carboxy group. The protected amino alcohol **2** was subsequently activated with mesyl chloride and reacted with NaN_3 providing azide **3**. Staudinger reduction of azide **3** gave rise to the second building block – *N*-protected aminomethyl pyrrolidine (**4**).

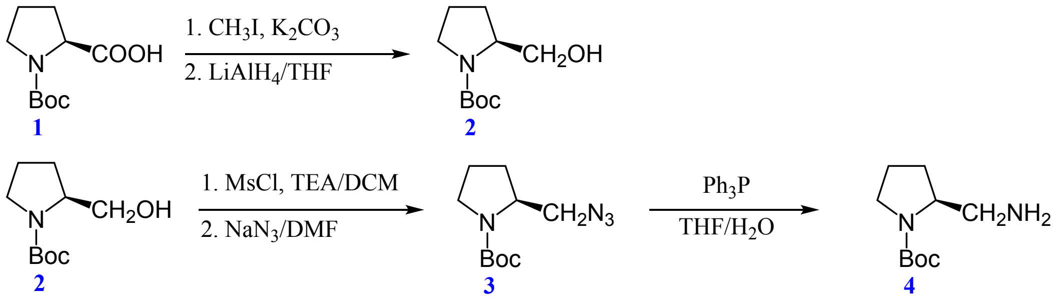

Scheme ESI-1. Synthesis of the pyrrolidine building blocks.

Acid azides **7a,b** were prepared by a sequence of reactions starting from methyl 3,5-dichloro-4-hydroxybenzoate (**5**), which was alkylated by propargyl bromide, or allyl bromide and then hydrolyzed by NaOH in aqueous ethanol yielding alkoxy acids **6a,b**.[S1] The acids **6a,b** were subsequently transformed to the corresponding acid chlorides by oxalyl chloride, which were reacted with aqueous solution of sodium azide to give the desired acid azides **7a,b** (**Scheme ESI-2**).

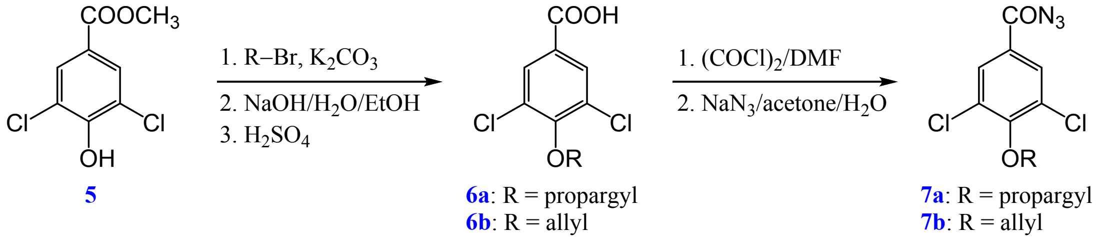

Scheme ESI-2. Synthesis of acid azides **7a,b**.

The adducts **9a-c** were prepared by the addition of **2** and **4**, resp., into a solution of the corresponding isocyanates (**8a,b**), which were obtained by *in situ* thermal decomposition of **7a,b** followed by Curtius rearrangement. In the case of **9a,b**, the reaction was catalyzed by dibutyl tin dilaurate (**Scheme ESI-3**).

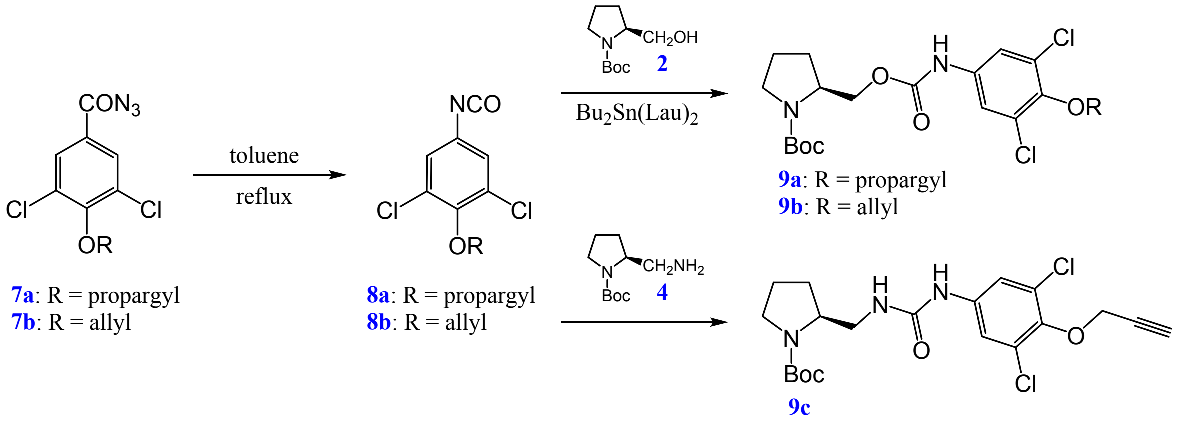

Scheme ESI-3. Synthesis of the precursors **9a-c**.

The remaining thiourea precursor **9d** was yielded from the *in situ* generated isocyanate **8a**, which was hydrolyzed with aq. KOH to afford aryl amine **10**. The amine **10** was transformed to the corresponding isothiocyanate **8c** by reaction with carbon disulphide in the presence of Boc anhydride and triethylamine.[S2] The key intermediate **9d** was subsequently obtained by a reaction with the mono-protected diamine **4** (**Scheme ESI-4**).

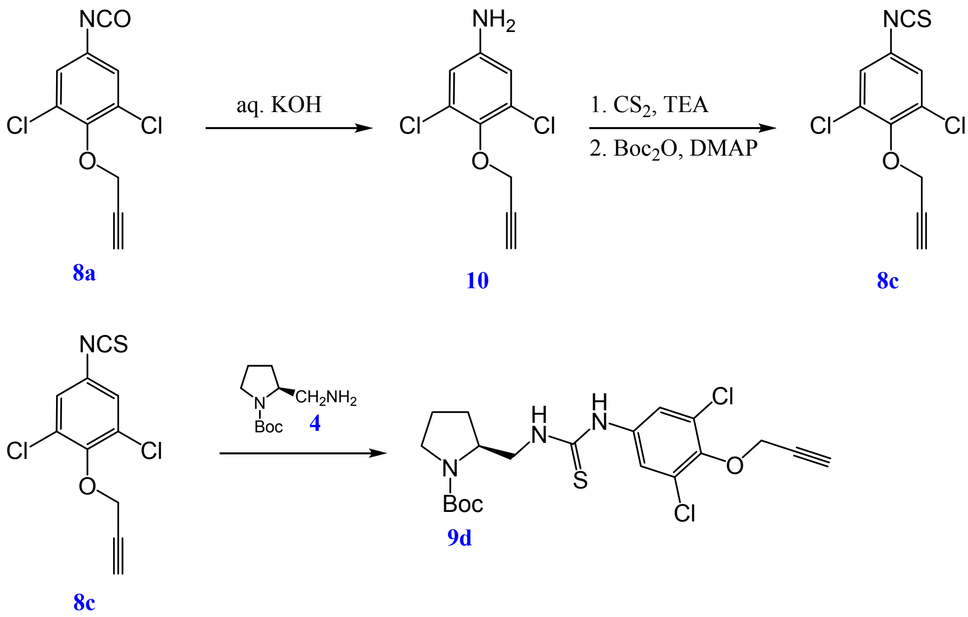

Scheme ESI-4. Synthesis of the precursor **9d**.

The precursors **9a-d** were deprotected with trifluoroacetic acid yielding the target compounds **Ia-d** (**Scheme ESI-5**).

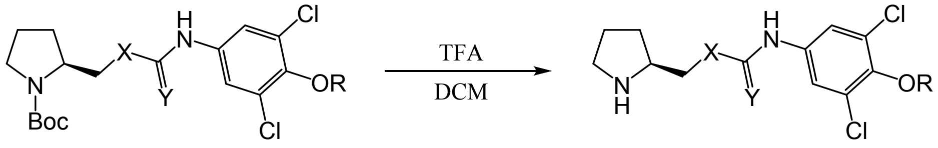

9a: X = O, Y = O, R = propargyl
9b: X = O, Y = O, R = allyl
9c: X = NH, Y = O, R = propargyl
9d: X = NH, Y = S, R = propargyl

Ia: X = O, Y = O, R = propargyl
Ib: X = O, Y = O, R = allyl
Ic: X = NH, Y = O, R = propargyl
Id: X = NH, Y = S, R = propargyl

Scheme ESI-5. Synthesis of the target compounds **Ia-d**.

3-Azidopropyl-modified silica (**11**) and 3-mercaptopropyl-modified silica (**12**) were prepared by previously reported procedures.[S3] Commercially available silica gel (5 μm particle size; 12 nm porosity; purchased from Daiso) was treated with 3-chloropropyl(trimethoxy)silane to give 3-chloropropyl-modified silica, which was transformed further by the means of NaN_3 yielding the desired azide-functionalized silica **11**. 3-Mercaptopropyl-modified silica (**12**) was yielded in a single-step reaction of the commercially available silica with 3-mercaptopropyl(methyl)dimethoxysilane.

The compounds **Ia,c,d** were immobilized to the solid phase by means of a Cu^{I}-catalyzed *click*-reaction with 3-azidopropyl-modified silica (**11**) giving rise to the target immobilized materials **IIa,c,d**. In the immobilization reaction of the amino thiourea **Id**, a stable complex with Cu^{I} was formed, thus preventing the catalysis of the reaction. Therefore, excess (1.2 equivalents) of the Cu^{I} catalyst was used to form the undesired complex quantitatively, thereby allowing the *click*-reaction to proceed with the excess of Cu^{I} present in the reaction mixture. The material **IIb** was yielded by radical addition of 3-mercaptopropyl-modified silica (**12**) to the corresponding compound **Ib** (**Scheme ESI-6**).

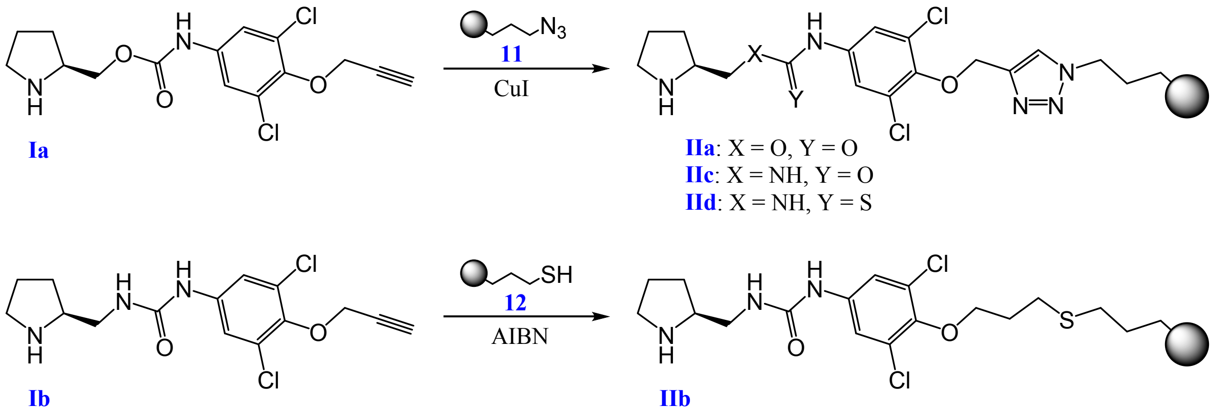

Scheme ESI-6. Synthesis of the target immobilized materials **IIa-d**.

Decomposition of Ic and IIc

Both amino urea compounds (catalysts) **Ic** and **IIc** provided only traces of the desired product in the model Michael addition. This fact can be ascribed to their low stability even at ambient temperature. We present a possible mechanism of the catalyst decomposition leading to its complete deactivation (**Scheme ESI-7**).

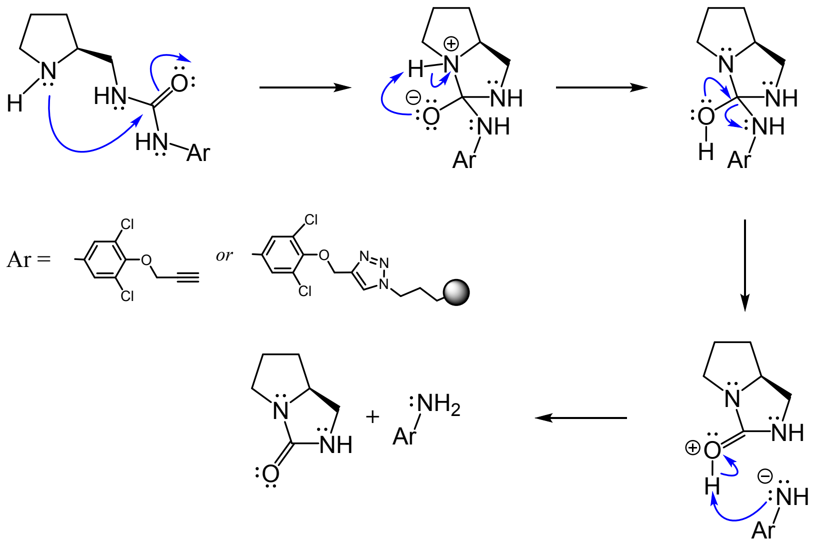

Scheme ESI-7. The mechanism of the catalysts decomposition.

As stated in the paper, the decomposition mechanism has been confirmed by treatment of **Ic** with ethanol at reflux. The expected decomposition products were isolated (see **Scheme 3** in the paper; for experimental procedure, see Experimental section, p. 23).

To explain why this decomposition takes place only in the case of the amino urea catalysts **Ic** and **IIc** and not the other catalysts, several aspects need to be considered. As described in **Scheme ESI-7**, the first step of the decomposition mechanism is a nucleophilic addition of the free amino group to the urea (carbamate, thiourea) moiety. From that point, there are three possible pathways for further transformations: (*a*) the backwards reaction leading to the active catalyst; (*b*) the exclusion of aryl amine leading to a bicyclic urea (carbamate, thiourea) product; (*c*) the exclusion of aliphatic amine (alcohol) leading to rearranged amino urea (hydroxy urea, amino thiourea) compound (see **Scheme ESI-8**).

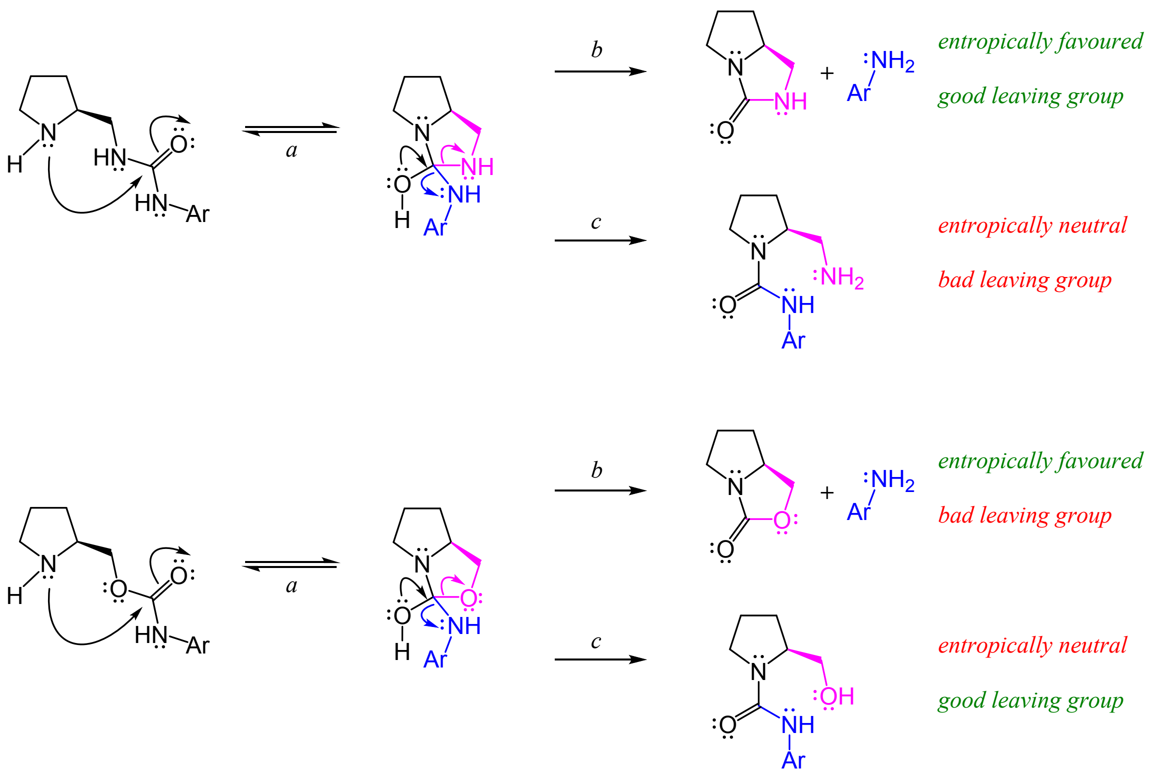

Scheme ESI-8. Possible reaction pathways of the catalyst decomposition.

In the case of amino urea catalysts **Ic** and **IIc**, the pathway (*b*) is preferred, since the arylamine functionality is a good leaving group in comparison to the aliphatic amine moiety. Moreover, the reaction provides a bicyclic product, which makes it entropically favoured. On the other hand, the pathway (*c*) seems improbable due to the worse leaving group and close to zero entropy factor.

In the case of amino carbamate catalysts **Ia,b** and **IIa,b**, both pathways (*b*) and (*c*) are partly disfavoured, either due to a bad leaving group (aryl amine vs. alcohol/alcoholate), or entropic factor, which explains the stability of the catalysts towards decomposition.

The catalysts **Id** and **IId** feature a less electrophilic thiourea moiety. Therefore, they are not prone towards the aforementioned decomposition.

It is reasonable to assume that in the case of other catalysts (**Ia,b,d**, **IIa,b,d**), the decomposition mechanism would take place as well at elevated reaction temperature. However, under the given reaction conditions the catalysts have proven to be stable.

Mechanism of the studied Michael addition

The studied catalyzed Michael addition of cyclohexanone and (*E*)-*β*-nitrostyrene can potentially lead to four different stereoisomers, each determined by its own diastereomeric transition state (**Scheme ESI-9**).

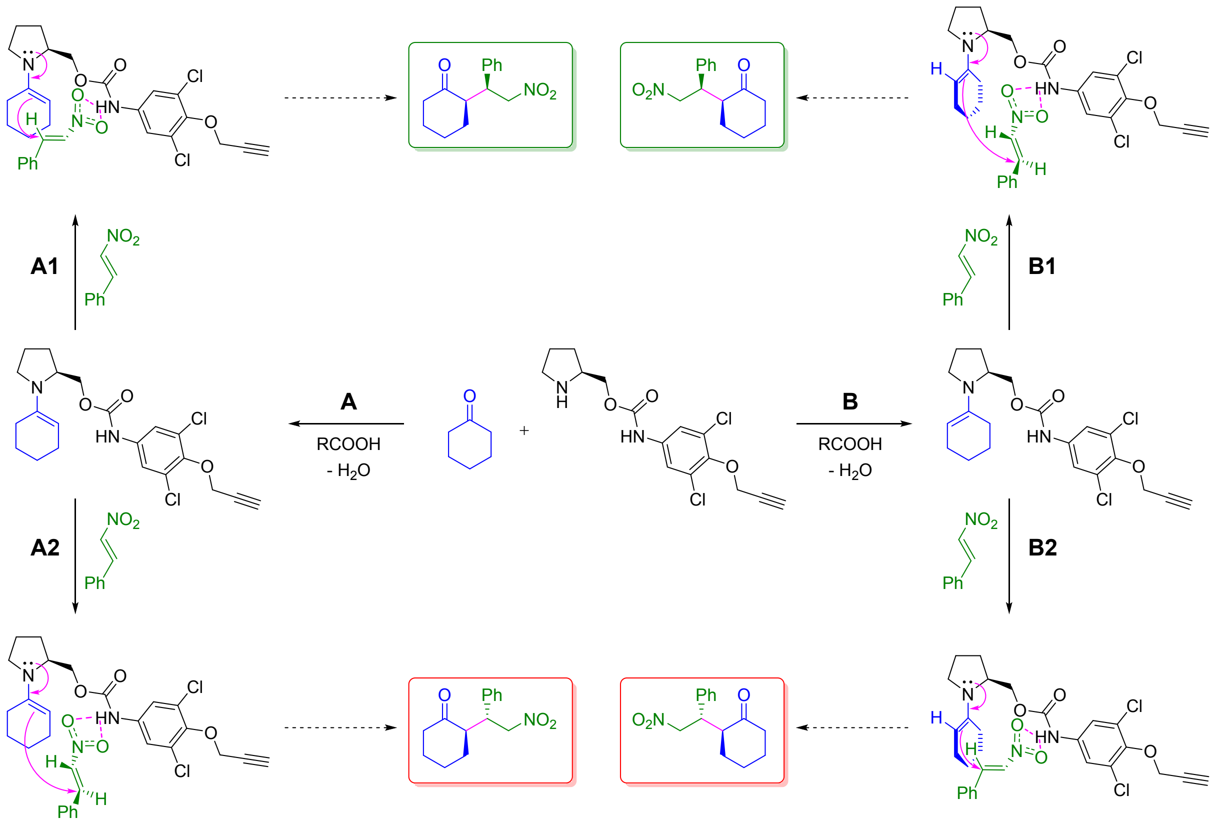

Scheme ESI-9. Proposed catalytic pathways towards all stereoisomers of the product.

First, enamine bond between the free secondary amino group of the catalyst and cyclohexanone is formed. The double bond of the enamine can be oriented either towards the lengthening arm of the catalyst (pathway **A**), or the opposite way (pathway **B**). Afterwards, (*E*)-*β*-nitrostyrene is coordinated to the lengthening arm of the catalyst via hydrogen bonding. In the case of pathways **A2** and **B2**, the mutual orientation of the reagents is disfavoured due to practically perpendicular orientation of the frontier orbitals (HOMO for enamine and LUMO for nitrostyrene). Therefore, the orbitals cannot interact profitably to form a new bond. On the other hand, the pathways **A1** and **B1** both provide parallel alignment of the reagents suitable for the ongoing reaction. Hence the generally high *syn*-selectivity found in the catalytic reactions (*syn*:*anti* up to 97:3).

The evaluation of the preference of the pathway **B1** over **A1** (leading towards opposite enantiomers of the *syn*-product) from the proposed reaction pathways would be overly speculative. It might be argued that there are sterical factors in place that determine the enantiomeric excess of the reaction. Further investigation, possibly via *ab-initio* calculations, would be required.

Experimental

Nuclear magnetic resonance measurements were performed on Agilent 400-MR DDR2 spectrometer operating at 400.13 MHz for ^{1}H and 100.62 MHz for ^{13}C. Chemical shifts are referenced internally to the residual non-deuterated solvent.

High-performance liquid chromatography measurements and catalytic experiments under continuous flow arrangement were performed using an 1100 Series HPLC system (Agilent Technologies).

Electronic circular dichroism measurements were carried out with a Jasco J-815 spectrometer, typically in the spectral range of 200-350 nm with scanning speed of 50 nm/min in a thin cell (cell length l = 1.0 mm) at sample concentration of 5 mg/ml.

(*S*)-*N*-*tert*-Butyloxycarbonyl-2-hydroxymethylpyrrolidine (2).

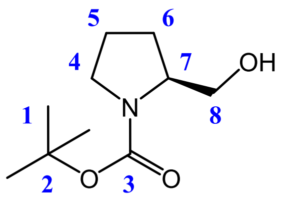

Methyl iodide (16.0 ml; 257 mmol) was added drop wise to a suspension of *N*-Boc-L-proline (30.0 g; 139 mmol) and anhydrydous K_2CO_3 (34.6 g; 250 mmol) in acetone (430 ml). The reaction mixture was stirred and heated to reflux in the absence of air moisture for 3 h. The reaction was quenched with water (100 ml) and the solvent was partly evaporated in vacuo to approximately 1/4 of its original volume. The residue was extracted with $CHCl_3$ (4×80 ml). Collected organic layers were washed with water (80 ml) and dried over $MgSO_4$. The resulting oil was diluted in dry THF (50 ml) and added drop wise to a suspension of $LiAlH_4$ (11.5 g; 303 mmol) in dry THF (250 ml) at 0 °C in argon atmosphere. The reaction mixture was stirred for 1 h, then the reaction was quenched with 1M aq. solution of KOH. The resulting solids were filtered and repeatedly washed with hot THF. The filtrate was extracted with $CHCl_3$ (4×50 ml). Collected organic layers were washed with an aq. solution of NH_4Cl and water (each 100 ml) and dried over $MgSO_4$. The solvent was evaporated and the crude product recrystallized from cyclohexanone to afford 20.8 g (74%) of white solid. m.p. 57-59 °C (ref.[S4] 57-60 °C).

^{1}H NMR ($CDCl_3$): 1.44 s, 9 H (H^{1}); 1.38-1.59 m, 1 H (H^{6a}); 1.68-1.79 m, 2 H ($H^{5a,b}$); 1.91-2.08 m, 1 H (H^{6b}); 3.22-3.34 m, 1 H (H^{4a}); 3.36-3.57 m, 1 H (H^{4b}); 3.49-3.66 m, 2 H ($H^{8a,b}$); 3.78-3.99 m, 1 H (H^{7}); 4.78 bs, 1 H (OH).

^{13}C NMR ($CDCl_3$): 24.3 (C^{5}); 28.7 (C^{1}); 28.9 (C^{6}); 47.8 (C^{4}); 60.4 (C^{7}); 67.8 (C^{8}); 80.4 (C^{2}); 154.7 (C^{3}).

NMR spectra correspond to the ref.[S4].

(*S*)-*N*-*tert*-Butyloxycarbonyl-2-azidomethylpyrrolidine (3).

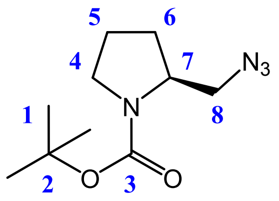

Mesyl chloride (1.16 ml; 15.0 mmol) was added to a solution of the hydroxy carbamate **2** (2.01 g; 10.0 mmol) and TEA (2.10 ml; 15.0 mmol) in dry DCM (30 ml) at 0 °C in argon atmosphere. The resulting yellow suspension was stirred at 0 °C for 10 minutes, then the cooling bath was removed and the suspension was further stirred for 3 h. The solvent was evaporated, the residue mixed with water (35 ml) and extracted with EtOAc (3×25 ml). Collected organic layers were washed with an aq. solution of $NaHCO_3$ and water (each 25 ml) and dried over $MgSO_4$. The solvent was evaporated, the residue diluted in dry DMF (20 ml) and the obtained solution heated to 60 °C. NaN_3 (1.95 g; 30.0 mmol) was added and the resulting suspension stirred at 60 °C in argon atmosphere for 2.5 days. The reaction mixture was cooled down to r.t., mixed with water (35 ml) and extracted with EtOAc (3×35 ml). Collected organic layers were washed with brine (4×25 ml) and dried over $MgSO_4$. The crude product was purified by column chromatography (eluent hexane:EtOAc, 85:15) yielding 1.68 g (74%) of a clear oil.

^{1}H NMR ($CDCl_3$): 1.45 s, 9 H (H^1); 1.76-2.04 m, 4 H ($H^{5a,b}$, $H^{6a,b}$); 3.22-3.63 m, 4 H ($H^{4a,b}$, $H^{8a,b}$); 3.80-3.99 m, 1 H (H^7).

^{13}C NMR ($CDCl_3$) – mixture of rotamers: 23.0, 23.9 ($^*C^5$); 28.4 (C^1); 28.6, 29.4 ($^*C^6$); 46.6, 47.0 ($^*C^4$); 52.7, 53.7 ($^*C^8$); 56.5 (C^7); 79.6, 79.9 ($^*C^2$); 154.2, 154.5 ($^*C^3$).

*doubled signal in the spectrum due to the presence of rotamers.

NMR spectra correspond to the ref.[S5].

(*S*)-*N*-*tert*-Butyloxycarbonyl-2-aminomethylpyrrolidine (4).

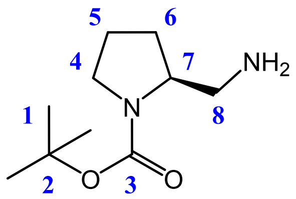

Triphenyl phosphane (1.92 g; 7.32 mmol) was slowly portion-wise added into a solution of the azido carbamate **3** (1.51 g; 6.67 mmol) in THF (12 ml) and water (1 ml). Gas evolution was observed. The resulting reaction mixture was heated to 45 °C and stirred overnight (18 h). The solvent was evaporated and the crude product was purified by column chromatography (eluent $CHCl_3$:MeOH:TEA, from 97:3:0.1 to 90:10:0.1) to yield 1.31 g (98%) of a yellowish oil.

^{1}H NMR ($CDCl_3$): 1.36 s 2 H (NH_2); 1.43 s, 9 H (H^1); 1.68-1.98 m, 4 H ($H^{5a,b}$, $H^{6a,b}$); 2.63 dd, 1 H (H^{8a}), ^2J = 12.91 Hz, ^3J = 7.04 Hz; 2.74-2.88 m, 1 H (H^{8b}); 3.24-3.49 m, 2 H ($H^{4a,b}$); 3.63-3.82 m, 1 H (H^7).

^{13}C NMR ($CDCl_3$) – mixture of rotamers: 23.1, 23.7 ($^*C^5$); 28.5 (C^1); 29.0, 29.6 ($^*C^6$); 45.4 (C^8); 46.6, 46.9 ($^*C^4$); 59.7 (C^7); 79.2 (C^2); 154.8, 155.2 ($^*C^3$).

*doubled signal in the spectrum due to the presence of rotamers. NMR spectra correspond to the ref.[S6].

3,5-Dichloro-4-prop-2-ynyloxybenzoic acid (6a).

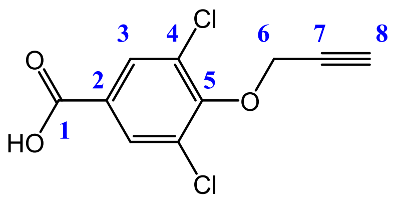

A solution of methyl 3,5-dichloro-4-hydroxybenzoate monohydrate (23.1 g; 96.6 mmol) in toluene (300 ml) was azeotropically distilled for 3.5 h. The solvent was evaporated and the residue diluted in acetone (250 ml). K_2CO_3 (18.0 g; 130 mmol) and propargyl bromide (14 ml of 80% wt. solution in toluene; 130 mmol) were added. The resulting mixture was stirred and heated to reflux in the absence of air moisture for 16 h. The reaction was quenched with water (200 ml) and the solvent was partly evaporated in vacuo to approximately 1/2 of its original volume. The reaction mixture was extracted with EtOAc (3×100 ml). Collected organic layers were washed with an aq. solution of $NaHCO_3$ and water (each 75 ml) and dried over $MgSO_4$. The solvent was evaporated and the residue was diluted in EtOH (100 ml). NaOH (5.8 g; 145 mmol) in water (100 ml) was added and the reaction mixture was stirred and heated to reflux for 30 min. The resulting mixture was cooled down to 0 °C and acidified with HCl (1:1 v/v) to *p*H = 1. The obtained precipitate was filtered and washed with water. Residual water from the crude product was removed by azeotropic distillation from toluene. The dehydrated product spontaneously crystallized from the toluene solution upon cooling to ambient temperature yielding 21.4 g (90%) of white crystals.

m.p. 202-206 °C.

^1H NMR (DMSO-d_6): 3.65 t, 1 H (H^8), ^4J = 2.35 Hz; 4.87 d, 2 H (H^6), ^4J = 2.35 Hz; 7.90 s, 2 H (H^3), 13.60 bs, 1 H (COOH).

^{13}C NMR (DMSO-d_6): 61.1 (C^6); 78.3 (C^7); 80.2 (C^8); 129.5 (C^4); 130.2 (C^3); 130.8 (C^5); 152.8 (C^2); 165.4 (C^1).

3,5-Dichloro-4-prop-2-enyloxybenzoic acid (6b).

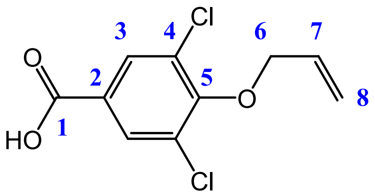

A solution of methyl 3,5-dichloro-4-hydroxybenzoate monohydrate (23.1 g; 96.6 mmol) in toluene (300 ml) was azeotropically distilled for 3.5 h. The solvent was evaporated and the residue diluted in acetone (250 ml). K_2CO_3 (50.0 g; 362 mmol) and allyl bromide (25 ml; 289 mmol) were added. The resulting mixture was stirred and heated to reflux in the absence of air moisture for 16 h. The reaction was quenched with water (200 ml) and the solvent was partly evaporated in vacuo to approximately 1/2 of its original volume. The reaction mixture was extracted with EtOAc (3×100 ml). Collected organic layers were washed with an aq. solution of $NaHCO_3$ and water (each 75 ml) and dried over $MgSO_4$. The solvent was evaporated and the residue was diluted in EtOH (100 ml). NaOH (5.8 g; 145 mmol) in water (100 ml) was added and the reaction mixture was stirred and heated to reflux for 30 min. The resulting mixture was cooled down to 0 °C and acidified with HCl (1:1 v/v) to *p*H = 1. The obtained precipitate was filtered and washed with water. Residual water from the crude product was removed by azeotropic distillation from toluene. The crude product was recrystallized from a toluene/ethanol mixture to give 18.7 g (63 %) of white needle-shaped crystals, m.p. 167-168 °C. ^{1}H NMR (acetone-d_6): 4.68 ddd, 2 H (H^6), ^3J = 5.86 Hz, ^4J = 3.22 Hz, ^4J = 2.64 Hz; 5.29 ddd, 1 H (H^{8cis}), ^2J = 1.46 Hz, ^3J = 10.54 Hz, ^4J = 2.64 Hz; 5.46 ddd, 1 H (H^{8trans}), ^2J = 1.46 Hz, ^3J = 17.28 Hz, ^4J = 3.22 Hz; 6.17 ddt, 1 H (H^7), ^3J = 5.86 Hz, ^3J = 10.54 Hz, ^3J = 17.28 Hz; 7.99 s, 2 H (H^3); 11.5 bs, 1 H (COOH).
^{13}C NMR (acetone-d_6): 74.4 (C^6); 118.3 (C^8); 128.0 (C^4); 129.5 (C^5); 130.2 (C^3); 132.9 (C^7); 154.8 (C^2); 164.1 (C^1).

3,5-Dichloro-4-(prop-2-ynyloxy)benzoyl azide (7a).

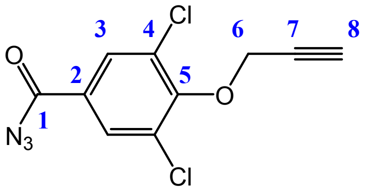

Acid **6a** (2.0 g; 8.16 mmol) was diluted in oxalyl chloride (20 ml) and a catalytic amount of DMF (50 µl) was added. The mixture was heated to reflux and stirred for 2 h. The excess of oxalyl chloride was distilled off. The residue was dissolved in hexane and treated with active charcoal for 2 min at boiling and then filtered while hot. The filtrate was evaporated; the formed acid chloride was diluted in acetone (35 ml) and cooled down to 0 °C. Then a solution of NaN_3 (530 mg; 8.16 mmol) in water (35 ml) was added drop wise. The reaction mixture was stirred at 0 °C for 1 h. The formed precipitate was filtered, repeatedly washed with an ice cold acetone/water = 4:1 mixture and subsequently diluted with dichloromethane (20 ml). Water (10 ml) was added and phases were separated. Aqueous layer

was extracted with DCM (3x5 ml) and the collected organic layers were dried over $MgSO_4$. Solvent was evaporated to yield 1.59 g (72%) of white solid.

m.p. 95-97 °C.

^{1}H NMR ($CDCl_3$): 2.55 t, 1 H (H^8), ^4J = 2.34 Hz; 4.88 d, 2 H (H^6), ^4J = 2.34 Hz; 7.97 s, 1 H (H^3).

^{13}C NMR ($CDCl_3$): 61.0 (C^6); 76.9 (C^8); 77.2 (C^7); 128.1 (C^4); 130.0 (C^3); 130.5 (C^5); 154.8 (C^2); 169.9 (C^1).

*MS (EI): for $C_{10}H_5Cl_2NO_2^+$ calculated: 240.9692; found: 240.9690.

*A peak of an ion formed after N_2 extrusion was detected rather than the molecular peak.

3,5-Dichloro-4-(prop-2-enyloxy)benzoyl azide (7b).

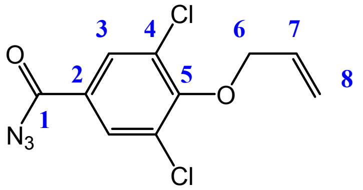

Acid **6b** (2.0 g; 8.10 mmol) was diluted in oxalyl chloride (20 ml) and a catalytic amount of DMF (50 µl) was added. The mixture was heated to reflux and stirred for 2.5 h. The excess of oxalyl chloride was distilled off. The residue was dissolved in hexane and treated with active charcoal for 2 min at boiling and then filtered while hot. The filtrate was evaporated; the formed acid chloride was diluted in acetone (35 ml) and cooled down to 0 °C. Then a solution of NaN_3 (520 mg; 8.00 mmol) in water (35 ml) was added drop wise. The reaction mixture was stirred at 0 °C for 1 h. The formed precipitate was filtered, repeatedly washed with an ice cold acetone/water = 4:1 mixture and subsequently diluted with dichloromethane (20 ml). Water (10 ml) was added and phases were separated. Aqueous layer was extracted with DCM (3x5 ml) and the collected organic layers were dried over $MgSO_4$. Solvent was evaporated to yield 1.40 g (64%) of white solid.

m.p. 46-47 °C.

^{1}H NMR ($CDCl_3$): 4.65 ddd, 2 H (H^6), ^3J = 5.86 Hz, ^4J = 3.22 Hz, ^4J = 2.64 Hz; 5.30 ddd, 1 H (H^{8cis}), ^2J = 1.46 Hz, ^3J = 10.25 Hz, ^4J = 2.64 Hz; 5.43 ddd, 1 H (H^{8trans}), ^2J = 1.46 Hz, ^3J = 17.28 Hz, ^4J = 3.22 Hz; 6.13 ddt, 1 H (H^7), ^3J = 5.86 Hz, ^3J = 10.25 Hz, ^3J = 17.28 Hz; 7.97 s, 2 H (H^3).

^{13}C NMR ($CDCl_3$): 74.7 (C^6); 119.5 (C^8); 127.5 (C^4); 130.0 (C^3); 130.2 (C^5); 132.3 (C^7); 156.1 (C^2); 170.0 (C^1).

*MS (EI): for $C_{10}H_7Cl_2NO_2^+$ calculated: 242.9854; found: 242.9859.

*A peak of an ion formed after N_2 extrusion was detected rather than the molecular peak.

3,5-Dichloro-4-prop-2-ynyloxyphenylamine (10)

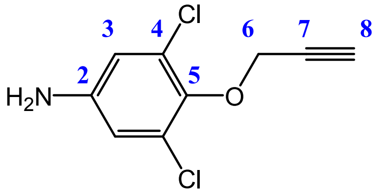

The acid azide **7a** (2.0 g; 7.41 mmol) was refluxed in dry toluene (70 ml) for 1 h, then the solvent was evaporated. The residue was dissolved in EtOH (70 ml) and heated to reflux. KOH (7.0 g) in water (35 ml) was added at once. The resulting solution was refluxed for 2.5 h, then cooled down to ambient temperature. The reaction mixture was extracted with EtOAc (3×50 ml). Collected organic layers were washed with aq. solution of NH_4Cl and H_2O (each 80 ml) and dried over $MgSO_4$. The crude product was purified by column chromatography (eluent hexane/ethyl acetate, from 9/1 to 4/1) and by recrystallization from cyclohexane to give 1.33 g (83%) of yellowish crystals.

m.p. 88-89 °C.

^{1}H NMR ($CDCl_3$): 2.52 t, 1 H (H^8), ^{4}J = 2.34 Hz; 3.65 bs, 2 H (NH_2); 4.66 d, 2 H (H^6), ^{4}J = 2.34 Hz; 6.60 s, 2 H (H^3).

^{13}C NMR ($CDCl_3$): 60.6 (C^6); 75.7 (C^8); 77.0 (C^7); 114.8 (C^3); 129.9 (C^4); 142.0 (C^5); 143.9 (C^2).

MS (ESI+): for $C_9H_8Cl_2NO^+$ calculated: 215.9978; found: 215.9972.

(*S*)-*O*-(*1-tert*-butyloxycarbonylpyrrolidin-2-yl)methyl *N*-(3,5-dichloro-4-prop-2-ynyloxyphenyl) carbamate (9a).

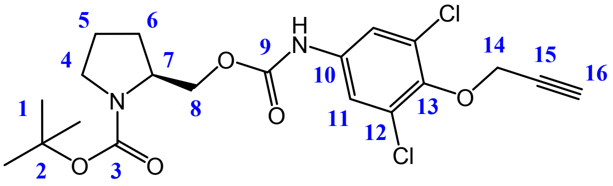

Azide **7a** (1.00 g; 3.70 mmol) was refluxed in dry toluene (40 ml) for 1 h. The reaction mixture was cooled down to 0 °C, then the protected amino alcohol **2** (625 mg; 3.10 mmol) in dry toluene (5 ml) was added drop wise along with dibutyl tin dilaurate (80 µl; 0.128 mmol; 95% purity). Resulting solution was stirred at 0 °C for 1 h in argon atmosphere, then the temperature was allowed to rise to room temperature and the solution was further stirred for 2.5 h. The solvent was evaporated and the crude product was purified by column chromatography (eluent hexane/ethyl acetate, 4/1) yielding 1.30 g (95%) of **9a** as highly viscous oil.

^{1}H NMR ($CDCl_3$): 1.46 s, 9 H (H^1); 1.81-2.03 m, 4 H ($H^{5a,b}$, $H^{6a,b}$); 2.52 t, 1 H (H^{16}), ^{4}J = 2.35 Hz; 3.24-3.49 m, 2 H ($H^{4a,b}$); 3.97-4.24 m, 3 H (H^7, $H^{8a,b}$); 4.71 d, 2 H (H^{14}), ^{4}J = 2.35 Hz; 7.43 bs, 2 H (H^{11}); 7.77 bs, 1 H (CO–NH).

^{13}C NMR ($CDCl_3$) – mixture of rotamers: 23.0, 23.6 ($^*C^5$); 27.7, 28.7 ($^*C^6$); 28.4 (C^1); 46.5 (C^4); 55.6 (C^7); 60.5 (C^{14}); 65.5, 65.8 ($^*C^8$); 76.1 (C^{16}); 77.9 (C^{15}); 79.9 (C^2); 118.5 (C^{11}); 129.7 (C^{12}); 136.0 (C^{13}); 145.2 (C^{10}); 153.5 (C^9); 155.2 (C^3).

*doubled signal due to presence of rotamers

MS (ESI+): for $C_{20}H_{24}Cl_2N_2NaO_5^+$ calculated: 465.0955; found: 465.0957.

(*S*)-*O*-(*1-tert*-butyloxycarbonylpyrrolidin-2-yl)methyl *N*-(3,5-dichloro-4-prop-2-enyloxyphenyl) carbamate (9b).

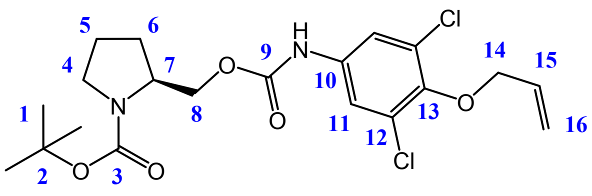

Azide **7b** (500 mg; 1.84 mmol) was refluxed in dry toluene (20 ml) for 1.5 h. The reaction mixture was cooled down to 0 °C, then the protected amino alcohol **2** ((310 mg; 1.54 mmol) in dry toluene (5 ml) was added drop wise along with dibutyl tin dilaurate (40 µl; 0.064 mmol; 95% purity). Resulting solution was stirred at 0 °C for 10 min in argon atmosphere, then the temperature was allowed to rise to room temperature and the solution was further stirred for 75 min. The solvent was evaporated and the crude product was purified by column chromatography (eluent hexane/ethyl acetate, 4/1) yielding 730 mg (89%) of **9b** as highly viscous oil.

^1H NMR ($CDCl_3$): 1.46 s, 9 H (H^1); 1.80-2.07 m, 4 H ($H^{5a,b}$, $H^{6a,b}$); 3.28-3.49 m, 2 H ($H^{4a,b}$); 3.97-4.26 m, 3 H (H^7, $H^{8a,b}$); 4.54 d, 2 H ($H^{14a,b}$), ^3J = 5.87 Hz; 5.26 dd, 1 H (H^{16cis}), ^2J = 1.56 Hz, ^3J = 10.56 Hz; 5.40 dd, 1 H ($H^{16trans}$), ^2J = 1.56 Hz, ^3J = 17.21 Hz; 6.12 ddt, 1 H (H^{15}), ^3J = 5.87 Hz, ^3J = 10.56 Hz, ^3J = 17.21 Hz; 7.45 s, 2 H (H^{11}); 7.47 bs, 1 H (CO-NH).

^{13}C NMR ($CDCl_3$) – mixture of rotamers: 23.1, 23.5 ($^*C^5$); 27.7, 29.7 ($^*C^6$); 28.5 (C^1); 46.5 (C^4); 55.5, 55.7 ($^*C^7$); 65.1, 65.7 ($^*C^8$); 74.4 (C^{14}); 79.9 (C^2); 118.8 (C^{16}); 119.0 (C^{11}); 129.6 (C^{12}); 133.0 (C^{15}); 135.1 (C^{13}); 146.7 (C^{10}); 153.2 (C^9); 154.7 (C^3).

*doubled signal in the spectrum due to the presence of rotamers

MS (ESI+): for $C_{20}H_{26}Cl_2N_2NaO_5^+$ calculated: 467.1111; found: 467.1112.

(*S*)-*N*-(*1-tert*-butyloxycarbonylpyrrolidin-2-yl)methyl *N′*-(3,5-dichloro-4-prop-2-ynyloxyphenyl) urea (9c).

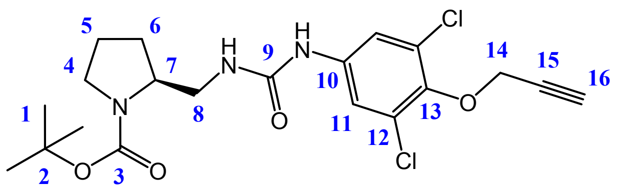

Azide **7a** (1.00 g; 3.70 mmol) was refluxed in dry toluene (40 ml) for 1 h. The mixture was cooled down to 0 °C, then the protected diamine **4** (620 mg; 3.10 mmol) in dry toluene (5 ml) was added drop wise. Resulting solution was stirred at 0 °C for 10 min in argon atmosphere, then the temperature was increased to room temperature and the solution was further stirred for 1 h. The solvent was evaporated and the crude product was purified by column chromatography (eluent hexane/ethyl acetate, from 2/1 to 1/1) yielding 1.06 g (77%) of **9c** as a white solid.

m.p. 151-152 °C.

^{1}H NMR (DMSO-d$_6$) – mixture of rotamers: 1.39 s, 9 H (H^{1}); 1.64-1.89 m, 4 H (H^{5a,b}, H^{6a,b}); 3.01-3.16 m, 1 H (H^{8a}); 3.18-3.36 m, 3 H (^{H4a,b}, H^{8b}); 3.59 t, 1 H (H^{16}), ^{4}J = 2.35 Hz; 3.70-3.79 m, 1 H (H^{7}); 4.69 d, 2 H (H^{14}), ^{4}J = 2.35 Hz; 6.39 s, 1 H (CO-NH^{1}); 7.49 s, 2 H (H^{11}); 8.68 s, 1 H (CO–NH^{2}), 8.78 s, 1 H (*CO–NH^{2}).

^{13}C NMR (DMSO-d$_6$) – mixture of rotamers: 22.9, 23.7 (*C^{5}); 28.3, 28.8 (*C^{6}); 28.6 (C^{1}); 41.8 (C^{8}); 46.7, 47.0 (*C^{4}); 57.2, 57.4 (*C^{7}); 60.9 (C^{14}); 78.8 (C^{16}); 78.9 (C^{2}); 79.6 (C^{15}); 117.8 (C^{11}); 128.9 (C^{12}); 138.8 (C^{13}); 143.3 (C^{10}); 154.0, 154.4 (*C^{3}); 155.3 (C^{9}).

*doubled signal due to the presence of rotamers

MS (ESI+): for $C_{20}H_{25}Cl_2N_3NaO_4^+$ calculated: 464.1114; found: 464.1116; a peak of 304.06177 (M+H$^+$ – C_3H_2) was also detected.

(*S*)-*N*-(*1-tert*-butyloxycarbonylpyrrolidin-2-yl)methyl *N′*-(3,5-dichloro-4-prop-2-ynyloxyphenyl) thiourea (9d).

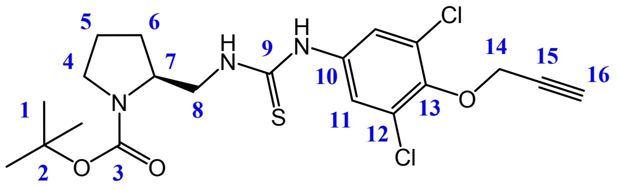

CS_2 (10.0 ml; 167 mmol) and TEA (1.2 ml; 8.6 mmol) were added into a solution of the aryl amine **10** (900 mg; 4.16 mmol) in anhydrous EtOH (5 ml; HPLC grade). The solution was stirred at ambient temperature in argon atmosphere for 48 h, while yellow suspension was formed. The suspension was cooled down to 0 °C, then Boc_2O (900 mg; 4.12 mmol) in anhydrous EtOH (5 ml; HPLC grade) was added drop wise followed by a catalytic amount of DMAP (10 mg; 0.08 mmol). The obtained mixture was stirred at 0 °C for 10 min, then at ambient temperature until the suspension cleared (2 h). The solvent was removed in

vacuo and the residue diluted in dry DCM (10 ml). The protected diamine **4** (690 mg; 3.45 mmol) in dry DCM (3 ml) was added drop wise. The resulting solution was stirred at r.t. in argon atmosphere for 30 min, then the solvent was evaporated. The crude product was purified by column chromatography (eluent hexane/ethyl acetate, from 6/1 to 3/1) to give 1.21 g (76%) of **9d** as a white solid.

m.p. 57-59 °C.

^{1}H NMR (DMSO-d$_6$; t = 60 °C): 1.40 s, 9 H (H^{1}); 1.71-1.93 m, 4 H ($H^{5a,b}$, $H^{6a,b}$); 3.22-3.34 m, 2 H ($H^{4a,b}$); 3.46-3.57 m, 2 H (H^{8a}, H^{16}); 3.63-3.78 m, 1 H (H^{8b}); 3.90-3.98 m, 1 H (H^{7}); 4.75 d, 2 H ($H^{14a,b}$), ^{4}J = 2.35 Hz; 7.64 s, 2 H (H^{11}); 7.91 bs, 1 H (CS-NH^{1}); 9.52 bs, 1 H (CS–NH^{2}).

^{13}C NMR (DMSO-d$_6$; t = 60 °C): 23.4 (C^{5}); 28.7 (C^{1}); 28.9 (C^{6}); 46.8 (C^{4}); 47.0 (C^{8}); 56.6 (C^{7}); 61.0 (C^{14}); 78.7 (C^{2}); 79.2 (C^{15}); 79.5 (C^{16}); 123.4 (C^{11}); 128.5 (C^{12}); 137.8 (C^{13}); 146.0 (C^{10}); 154.4 (C^{3}); 181.5 (C^{9}).

MS (ESI+): for $C_{20}H_{25}Cl_2N_3NaO_3S^+$ calculated: 480.0886; found: 408.0889.

(*S*)-*O*-(pyrrolidin-2-yl)methyl *N*-(3,5-dichloro-4-prop-2-ynyloxyphenyl) carbamate (Ia).

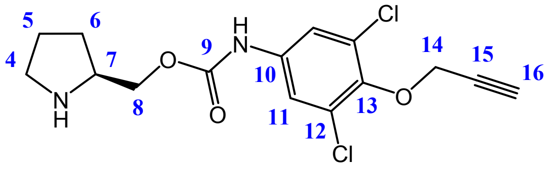

Trifluoroacetic acid (25 ml) was added drop wise into a solution of carbamate **9a** (1.30 g; 2.93 mmol) in dry dichloromethane (25 ml) at 0 °C in argon atmosphere. Resulting solution was stirred at 0 °C for 10 min, then the temperature was allowed to reach room temperature and the solution was further stirred for 40 min. The solvent was evaporated, the residue was mixed with water (20 ml), *p*H was adjusted with aq. $NaHCO_3$ to 8.5, the mixture was sonicated for 5 min and then extracted with dichloromethane (3×20 ml). Collected organic layers were washed with aq. $NaHCO_3$ and water (both 30 ml) and dried over $MgSO_4$. The crude product was purified by column chromatography (eluent dichloromethane/methanol/triethylamine, from 90/10/0.1 to 80/20/0.1) to give 720 mg (72%) of **Ia** as a yellow solid.

m.p. 83-87 °C.

^{1}H NMR (DMSO-d$_6$): 1.32-1.41 m, 1 H (H^{6a}); 1.55-1.72 m, 2 H ($H^{5a,b}$); 1.73-1.83 m, 1 H (H^{6b}); 2.48 s, 1 H (NH); 2.75-2.84 m, 2 H ($H^{4a,b}$); 3.27-3.33 m, 1 H (H^{7}); 3.59 t, 1 H (H^{16}), ^{4}J = 2.35 Hz; 3.89-3.98 m, 2 H ($H^{8a,b}$); 4.71 d, 2 H (H^{14}), ^{4}J = 2.35 Hz; 7.68 s, 2 H (H^{11}); 9.96 bs, 1 H (CO–NH).

^{13}C NMR (DMSO-d$_6$): 25.4 (C^5); 28.5 (C^6); 46.4 (C^4); 56.8 (C^7); 60.9 (C^{14}); 68.2 (C^8); 78.7 (C^{15}); 79.7 (C^{16}); 118.4 (C^{11}); 129.1 (C^{12}); 137.5 (C^{13}); 144.4 (C^{10}); 153.8 (C^9).

MS (ESI+): for $C_{15}H_{17}Cl_2N_2O_3^+$ calculated: 343.0611; found: 343.0616.

CD spectrum

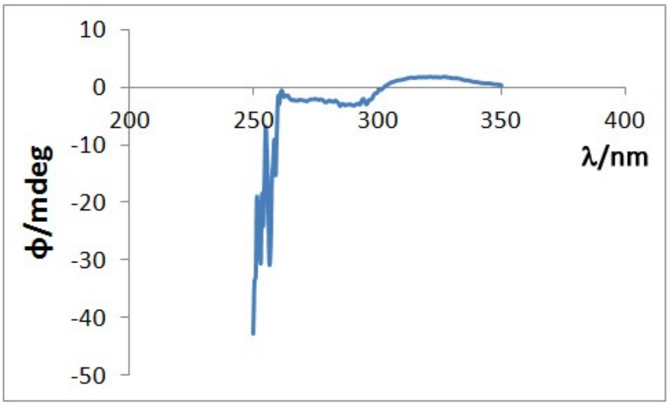

(*S*)-*O*-(pyrrolidin-2-yl)methyl *N*-(3,5-dichloro-4-prop-2-enyloxyphenyl) carbamate (Ib).

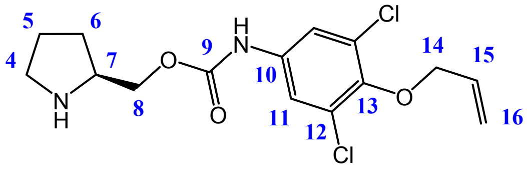

Trifluoroacetic acid (20 ml) was added drop wise into a solution of carbamate **9b** (720 mg; 1.62 mmol) in dry dichloromethane (20 ml) at 0 °C in argon atmosphere. Resulting solution was stirred at 0 °C for 10 min, then the temperature was allowed to reach room temperature and the solution was further stirred for 90 min. The residue was mixed with water (20 ml), alkalized with an aq. solution of $NaHCO_3$ to *p*H = 8.5, sonicated for 5 min and then extracted with DCM (4×30 ml). Collected organic layers were dried over $MgSO_4$. The crude product was purified by column chromatography (eluent chloroform/methanol/triethylamine, 90/10/0.1) to give 298 mg (54%) of **Ib** as yellowish solid.

m.p. 79-82 °C.

^1H NMR ($CDCl_3$): 1.39-1.48 m, 1 H (H^{6a}); 1.70-1.86 m, 2 H ($H^{5a,b}$); 1.86-1.97 m, 1 H (H^{6b}); 2.70 bs, 1 H (NH); 2.90-3.05 m, 2 H ($H^{4a,b}$); 3.39-3.48 m, 1 H (H^7); 4.03 dd, 1 H (H^{8a}), ^2J = 10.95 Hz, ^3J = 8.61 Hz; 4.17 dd, 1 H (H^{8b}), ^2J = 10.95 Hz, ^3J = 3.91 Hz; 4.49 d, 2 H ($H^{14a,b}$), ^3J = 5.87 Hz; 5.26 dd, 1 H (H^{16cis}), ^2J = 1.17 Hz, ^3J = 10.56 Hz; 5.39 dd, 1 H ($H^{16trans}$), ^2J = 1.17 Hz, ^3J = 17.22 Hz; 6.11 ddt, 1 H (H^{15}), ^3J = 5.87 Hz, ^3J = 10.56 Hz, ^3J = 17.22 Hz; 7.35 s, 2 H (H^{11}); 8.19 bs, 1 H (CO-NH).

^{13}C NMR ($CDCl_3$): 25.3 (C^5); 27.8 (C^6); 46.3 (C^4); 57.3 (C^7); 67.9 (C^8); 74.4 (C^{14}); 118.7 (C^{16}); 118.8 (C^{11}); 129.6 (C^8); 133.0 (C^{15}); 135.1 (C^7); 146.7 (C^{10}); 153.5 (C^9).

MS (ESI+): for $C_{15}H_{19}Cl_2N_2O_3^+$ calculated: 345.0767; found: 345.0771.

CD spectrum:

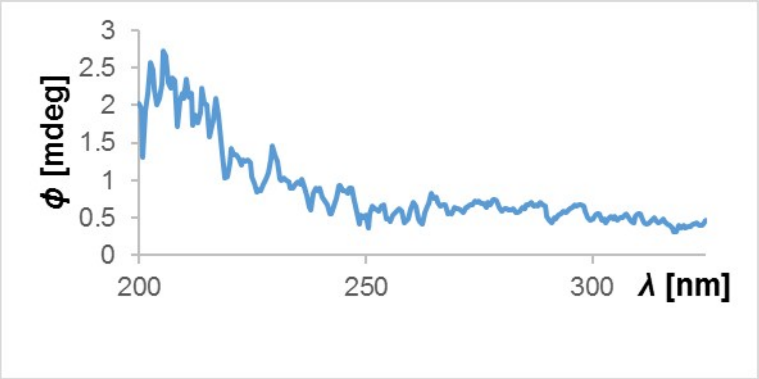

(*S*)-*N*-(pyrrolidin-2-yl)methyl *N′*-(3,5-dichloro-4-prop-2-ynyloxyphenyl) urea (Ic).

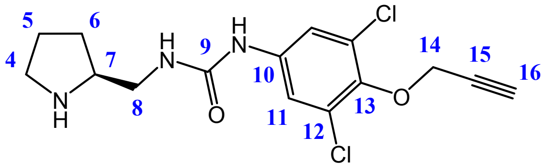

Trifluoroacetic acid (16 ml) was added drop wise into a solution of urea **9c** (900 mg; 2.03 mmol) in dry dichloromethane (16 ml) at 0 °C in argon atmosphere. The mixture was stirred at 0 °C for 5 min, then the temperature was allowed to reach room temperature and the solution was further stirred for 30 min. The solvent was evaporated, the residue was mixed with water (20 ml), *p*H was adjusted to 8.5 with aq. $NaHCO_3$, the mixture was sonicated for 5 min and then extracted with dichloromethane (3×20 ml). Collected organic layers were washed with aq. $NaHCO_3$ and water (both 20 ml) and dried over $MgSO_4$. The crude product was purified by column chromatography (eluent dichloromethane/methanol/triethylamine, from 90/10/0.1 to 50/50/0.2) to give 375 mg (54%) of **Ic** as a yellow solid.

m.p. 85-88 °C.

^{1}H NMR (DMSO-d$_6$) – mixture of tautomers: 1.26-1.40 m, 1 H (H^{6a}); 1.54-1.79 m, 3 H ($H^{5a,b}$, H^{6b}); 2.78 t, 2 H ($H^{4a,b}$), J = 6.65 Hz; 2.94-3.02 m, 1 H (H^{8a}); 3.04-3.16 m, 2 H (H^{7}, H^{8b}); 3.58 t, 1 H (H^{16}), ^{4}J = 2.35 Hz; 3.70 bs, 1 H (NH); 4.68 d, 2 H (H^{14}), ^{4}J = 2.35 Hz; 6.15 t, 1 H (CO–NH^{1}), J = 5.48 Hz; 6.57 t, 1 H (*CO-NH^{1}), J = 5.48 Hz; 7.15 s, 1 H (H^{11}); 7.49 s, 1 H ($^{*}H^{11}$); 8.37 bs, 1 H (CO–NH^{2}); 9.23 bs, 1 H (*CO–NH^{2}).

^{13}C NMR (DMSO-d$_6$) – mixture of tautomers: 25.5, 25.6 ($^{*}C^{5}$); 28.9, 29.0 ($^{*}C^{6}$); 44.0 (C^{8}); 46.1, 46.2 ($^{*}C^{4}$); 58.3, 58,7 ($^{*}C^{7}$); 60.2, 60.8 ($^{*}C^{14}$); 78.8 (C^{15}); 79.5 (C^{16}); 117.7 (C^{11}); 128.9 (C^{12}); 139.1 (C^{13}); 143.2 (C^{10}); 155.6, 156.4 ($^{*}C^{9}$).

*doubled signal in the spectrum due to tautomerization

MS (ESI+): for $C_{15}H_{18}Cl_2N_3O_2^{+}$ calculated: 342.0771; found: 342.0776;

CD spectrum

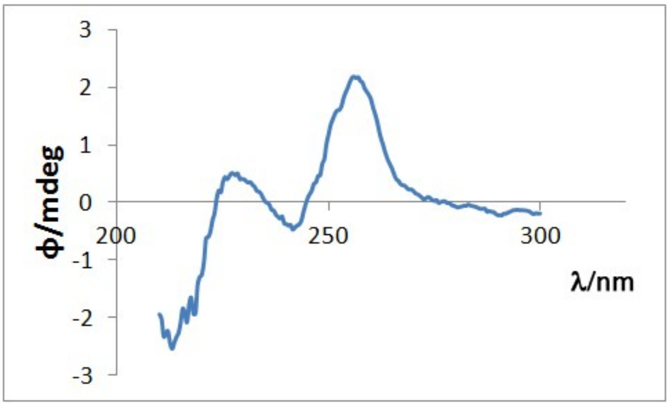

(*S*)-*N*-(pyrrolidin-2-yl)methyl *N´*-(3,5-dichloro-4-prop-2-ynyloxyphenyl) thiourea (Id).

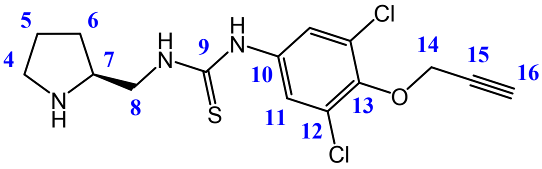

Trifluoroacetic acid (15 ml) was added drop wise into a solution of urea **9d** (1.18 g; 2.57 mmol) in dry dichloromethane (20 ml) at 0 °C in argon atmosphere. The mixture was stirred at 0 °C for 30 min, then the temperature was allowed to reach room temperature and the solution was further stirred for 90 min. The solvent was evaporated, the residue was mixed with water (20 ml), *p*H was adjusted to 8.5 with aq. $NaHCO_3$, the mixture was sonicated for 5 min and then extracted with chloroform (4×15 ml). Collected organic layers were washed with aq. $NaHCO_3$ and water (both 20 ml) and dried over $MgSO_4$. The crude product was purified by column chromatography (eluent chloroform/methanol/triethylamine, from 80/20/0.1 to 70/30/0.2) to give 783 mg (85%) of **Id** as a pink solid.

m.p. 83-84 °C (decomposition).

^{1}H NMR (DMSO-d$_6$; *t* = 80 °C): 1.34-1.46 m, 1 H (H^{6a}); 1.56-1.83 m, 3 H (H^{5a,b}, H^{6b}); 2.75-2.90 m, 2 H (H^{4a,b}); 3.26-3.37 m, 2 H (H^{7}, H^{8a}); 3.39-3.52 m, 2 H (H^{8b}, H^{16}); 4.75 d, 2 H (H^{14a,b}), ^{4}J = 2.35 Hz; 7.68 s, 2 H (H^{11}); all exchangable hydrogens (NH, CS-NH^{1}, CS-NH^{2}) exchanged with the solvent.

^{13}C NMR ((DMSO-d$_6$; *t* = 80 °C): 25.9 (C^{5}); 29.2 (C^{6}); 46.2 (C^{4}); 49.3 (C^{8}); 57.8 (C^{7}); 61.0 (C^{14}); 78.8 (C^{15}); 79.2 (C^{16}); 122.9 (C^{11}); 128.4 (C^{12}); 138.5 (C^{13}); 145.7 (C^{10}); 181.7 (C^{9}).

MS (ESI+): for $C_{15}H_{18}Cl_2N_3OS^+$ calculated: 358.0542; found: 358.0552.

CD spectrum:

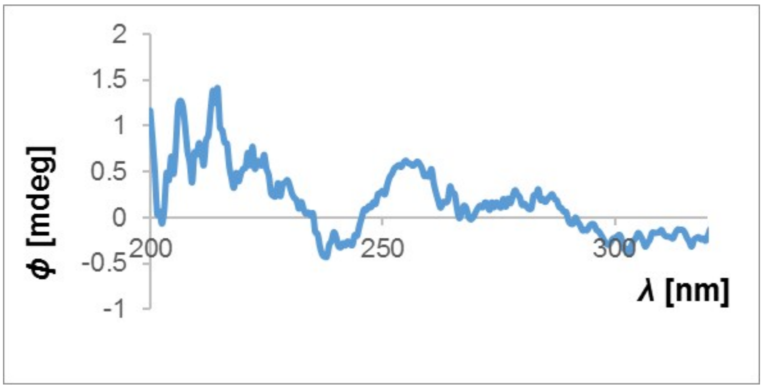

Immobilized amino carbamate IIa.

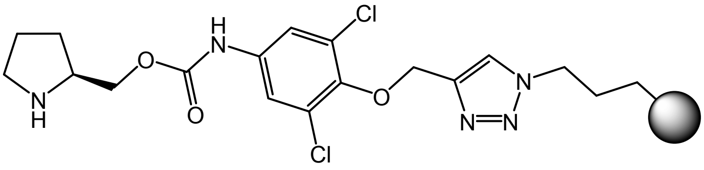

Amino carbamate **Ia** (236 mg; 688 µmol) was dissolved in dry acetonitrile (60 ml). Then azidopropyl-modified silica (**11**, 2.50 g), diisopropyl ethyl amine (240 µl; 1.40 mmol), and CuI (26 mg; 131 µmol) were added. The suspension was vigorously mechanically stirred in argon atmosphere for 3 days. The suspension was filtered and the silica washed with acetonitrile, methanol, 2% aq. ethylenediaminetetraacetic acid disodium salt, aqueous methanol (1:1), methanol and acetonitrile. The resulting catalyst **IIa** was dried under reduced pressure for 3 days.

Elemental analysis: It was found 7.92% C, 1.38% H and 3.84% N, which corresponds to a coverage (loading) of 230 µmol/g.

Immobilized amino carbamate IIb.

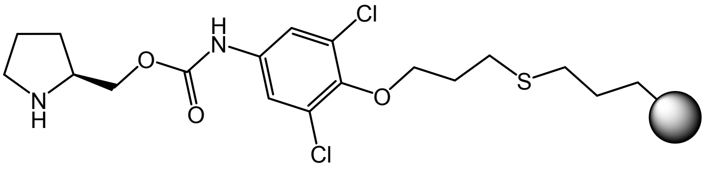

The amino carbamate **Ib** (260 mg; 753 µmol) in anhydrous MeOH (5 ml; HPLC grade) and AIBN (25 mg; 152 µmol) were added into a slurry of mercaptopropyl-modified silica (**12**, 2.50 g) in anhydrous MeOH (15 ml; HPLC grade) in an argon-flushed three-neck flask. The obtained suspension was heated to 75 °C and vigorously mechanically stirred for 3.5 h in argon atmosphere. Afterwards, another portion of AIBN (25 mg; 152 µmol) was added. The suspension was further stirred for 4 h, then filtered. The silica was repeatedly washed with MeOH and dried in vacuo for 3 days.

Elemental analysis: It was found 4.98% C, 1.15% H, 0.70% N and 1.97% S, which corresponds to a coverage (loading) of 80 μmol/g.

Immobilized amino urea IIc.

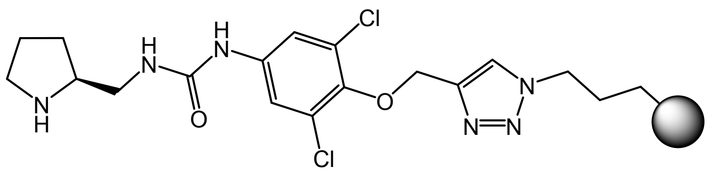

As for **Ia**, the mixture of amino urea **Ic** (236 mg; 688 μmol) azidopropyl-modified silica (**11**, 2.50 g), diisopropyl ethyl amine (240 μl; 1.40 mmol), and CuI (26 mg; 131 μmol) in acetonitrile was vigorously mechanically stirred in argon atmosphere for 3 days. The suspension was filtered, washed and dried under reduced pressure for 3 days providing the heterogeneous catalyst **IIc**.

Elemental analysis: It was found 8.27% C, 1.53% H and 4.35% N, which corresponds to a coverage (loading) of 249 μmol/g.

Immobilized amino thiourea IId.

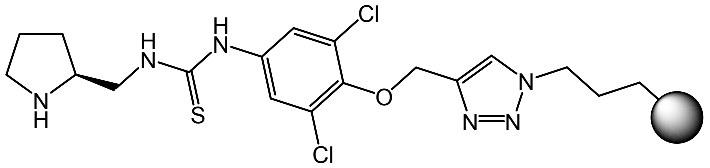

The amino thiourea **Id** (250 mg; 698 μmol) was diluted in dry DMF (65 ml) in a three-neck flask. Azidopropyl-modified silica (**11**, 2.50 g) and CuI (160 mg; 840 μmol) were added. The resulting suspension was vigorously mechanically stirred in argon atmosphere for 24 h. The suspension was filtered and the silica washed with DMF, water, 2% aq. EDTA, water, MeOH and Et_2O. The obtained product was dried in vacuum for 3 days.

Elemental analysis: It was found 6.97% C, 1.23% H, 3.74% N and 0.59% S, which corresponds to a coverage (loading) of 215 μmol/g.

Synthesis of the silica gel solid supports.

3-Azidopropyl-modified silica (11).

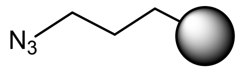

Silica (16.7 g; 5 μm size; 12 nm porosity) was suspended in toluene (250 ml) in a three-neck flask, which was subsequently washed with argon. The obtained suspension was heated to reflux and residual traces of water were removed by azeotropic distillation. The reaction mixture was cooled down to room temperature, and (3-chloropropyl)trimethoxysilane (25 ml; 178 mmol) and DMAP (25 mg; 0.204 mmol) were added at once. The resulting mixture was heated to 90 °C and stirred overnight (17 h). The

suspension was let cool down to ambient temperature and filtered. The modified silica was repeatedly washed with toluene (3×50 ml) and ethyl acetate (3×50 ml) and dried in vacuum for 3 days. The obtained 3-chloropropyl-modified silica (18.6 g) and tetrabutylammonium iodide (100 mg; 0.27 mmol) were added to a 0.5M solution of NaN_3 (16.0 g; 152.5 mmol) in anhydrous DMSO (120 ml) in a flame-dry three-neck flask at 90 °C in argon atmosphere. The suspension was stirred at 90 °C for 3 days, and then filtered. The obtained silica was repeatedly washed with water and MeOH and dried in vacuum for 3 days.
Elemental analysis: It was found 3.47% C, 0.88% H and 3.30% N, which corresponds to coverage (loading) of 785 μmol/g.

3-Mercaptopropyl-modified silica (12).

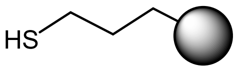

Silica (20.0 g; 5 μm size; 12 nm porosity) was suspended in toluene (250 ml) in a three-neck flask, which was subsequently washed with argon. The obtained suspension was heated to reflux and residual traces of water were removed by azeotropic distillation. The suspension was let cool down to ambient temperature and (3-mercaptopropyl)methyldimethoxysilane (6 ml; 48.2 mmol) and DMAP (38 mg; 0.31 mmol) were added at once. The resulting mixture was heated to reflux and mechanically stirred for 7 h. The suspension was left to cool down to ambient temperature and filtered. The solids were repeatedly washed with hot toluene and methanol and dried in vacuum for 3 days.
Elemental analysis: It was found 5.00% C, 1.25% H and 2.24% S, which corresponds to coverage (loading) of 698 μmol/g.

2-(2-Nitro-1-phenyl)ethylcyclohexanone (general procedure for catalytic reactions).[*]
Catalyst (0.05 mmol) was added to a solution of cyclohexanone (0.25 ml) and either butyric acid (0.0025 mmol), or acetic acid (0.035 mmol) of a chosen solvent (1 ml). The resulting mixture was stirred at ambient temperature for 15 minutes, then (*E*)-*β*-nitrostyrene (37.3 mg; 0.25 mmol) was added at once. The reaction mixture was stirred at either room temperature or 0 °C for 2-6 days. Afterwards, the solvent was thoroughly evaporated under reduced pressure and the crude product purified by flash column chromatography (eluent hexane/ethyl acetate, 5/1).
^{1}H NMR ($CDCl_3$): 1.17-1.30 m, 1 H (H^{3b}); 1.51-1.83 m, 4 H (H^{3b}, $H^{4a,b}$, H^{5a}); 2.03-2.13 m, 1 H (H^{5b}); 2.34-2.43 m, 1 H (H^{6a}); 1.44-2.52 m, 1 H (H^{6b}); 2.64-2.74 m, 1 H (H^{2}); 3.72-3.81 m, 1 H (H^{7}); 4.63 dd, 1 H (H^{8a}), ^2J = 12.13 Hz; ^3J = 10.17 Hz; 4.94 dd, 1 H (H^{8b}), ^2J = 12.13 Hz; ^3J = 3.52 Hz; 7.14-7.19 m, 2 H (H^{10}); 7.23-7.36 m, 3 H (H^{11}, H^{12}).

^{13}C NMR ($CDCl_3$): 25.0 (C^4); 28.5 (C^5); 33.2 (C^3); 42.7 (C^6); 43.9 (C^7); 52.5 (C^2); 78.9 (C^8); 127.8 (C^{10}); 128.2 (C^{12}); 128.9 (C^{11}); 137.7 (C^9); 211.9 (C^1).

*for specific reaction conditions see the footnotes of the tables listed in the paper

Decomposition study of Ic

Amino urea **Ic** (68.4 mg; 0.20 mmol) was dissolved in ethanol (10 ml). The reaction mixture was heated to reflux and stirred for 1.5 h. The solvent was evaporated and the residue purified by column chromatography (eluent hexane/ethyl acetate, from 6/1 to 3/1). 15.8 mg (63%) of (*S*)-tetrahydro-1H-pyrrolo[1,2-c]imidazol-3(2H)-one were isolated.

m.p. 178-179 °C (ref.[S7] 178-180 °C).

^{1}H NMR ($CDCl_3$): 1.28-1.39 m, 1 H (H^{3a}); 1.60-1.88 m, 3 H ($H^{2a,b}$, H^{3b}); 1.95 s, 3 H (H^7); 2.22 bs, 1 H (NH); 2.87 dd, 2 H ($H^{1a,b}$), ^3J = 7.04 Hz, ^3J = 6.65 Hz; 2.94-3.02 m, 1 H (H^{5a}); 3.18-3.26 m, 1 H (H^4); 3.33-3.41 m, 1 H (H^{5b}); 6.24 bs, 1 H (CO-NH). ^{13}C NMR ($CDCl_3$): 23.2 (C^7); 25.8 (C^2); 29.1 (C^3); 43.7 (C^5); 46.4 (C^1); 57.7 (C^4); 170.3 (C^6).

31.3 mg (72%) of 3,5-dichloro-4-prop-2-ynyloxyphenylamine (**10**) were also isolated.

m.p. 88-90 °C

NMR spectra are listed above (p. 13).

HPLC separation using stationary phases IIb-d

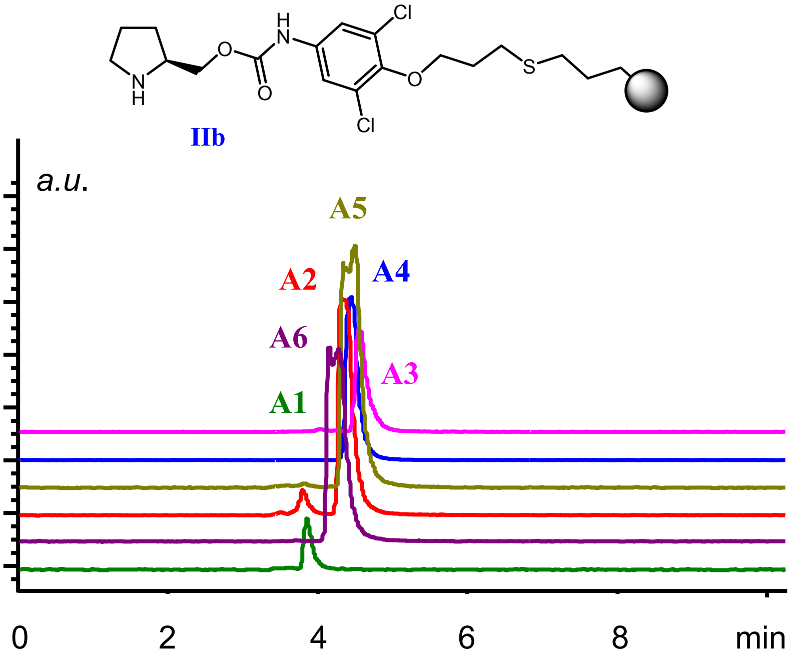

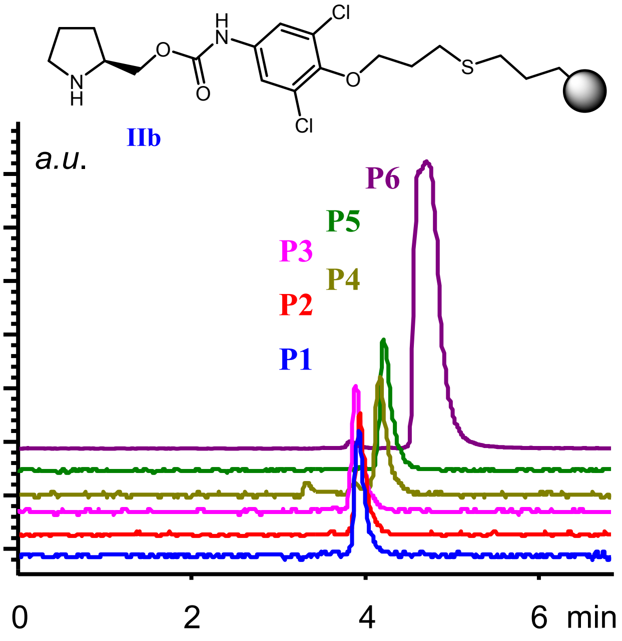

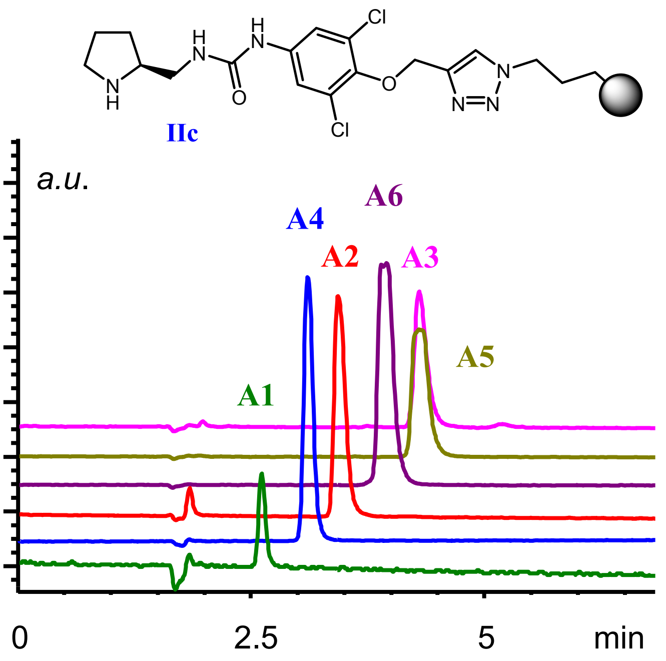
IIc
a.u.
A1
A2
A3
A4
A5
A6
0
2.5
5
min

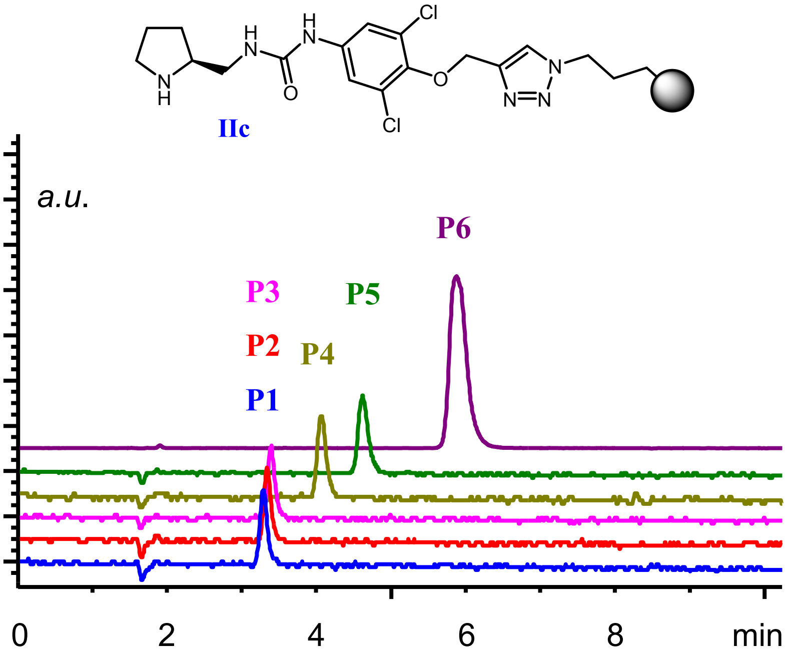
IIc
a.u.
P1
P2
P3
P4
P5
P6
0
2
4
6
8
min

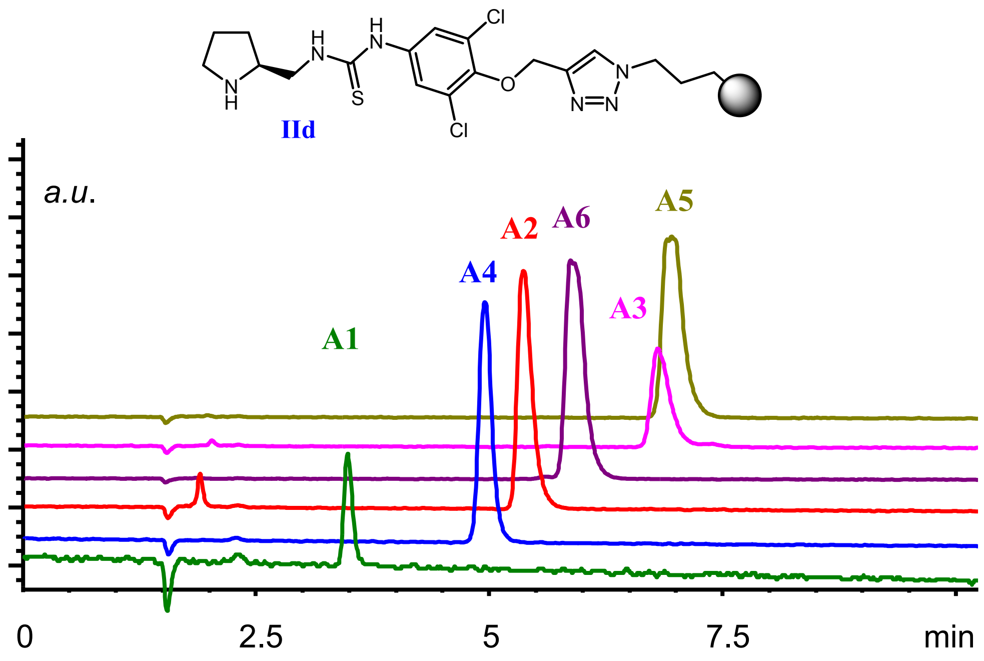
IId
a.u.
A1
A4
A2
A6
A3
A5
0
2.5
5
7.5
min

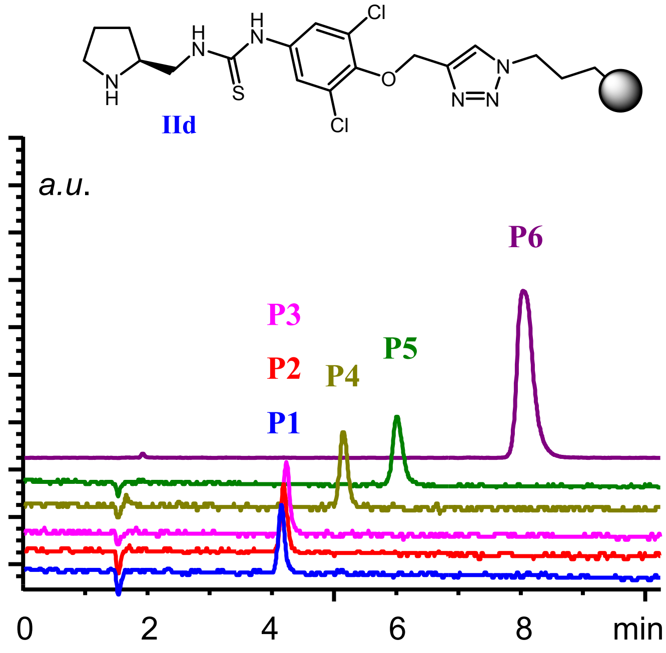
IId
a.u.
P3
P2
P1
P4
P5
P6
0
2
4
6
8
min

NMR spectra

^{1}H NMR; $CDCl_3$

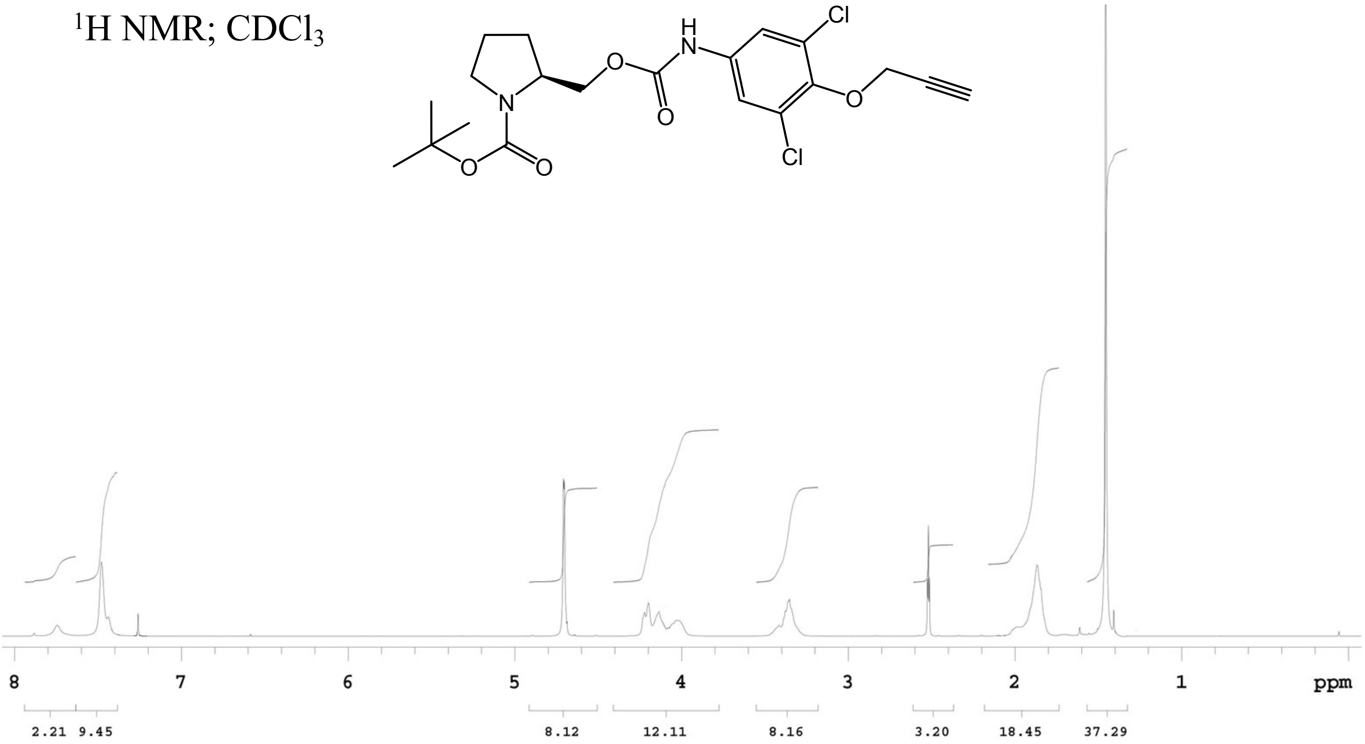

^{13}C NMR; $CDCl_3$

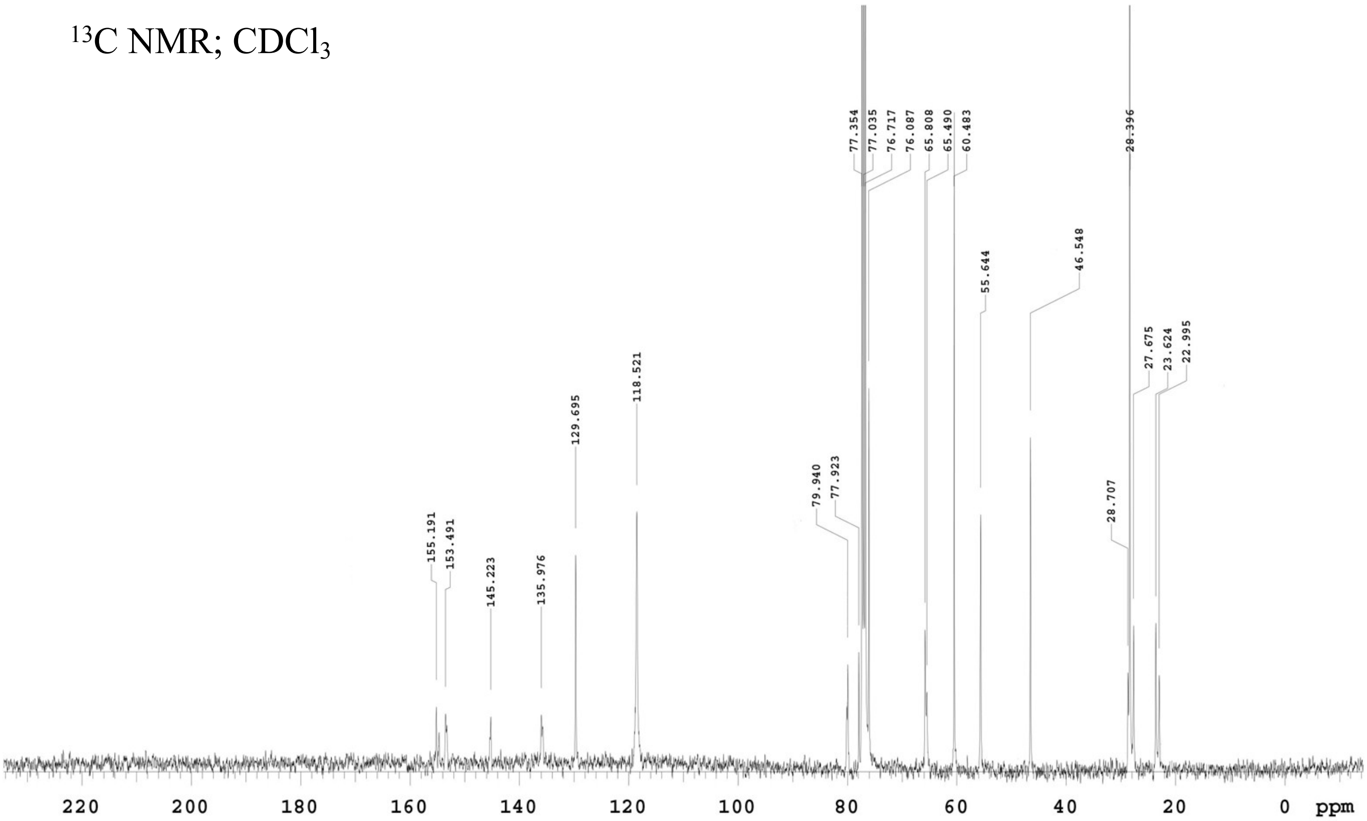

^{1}H NMR; $CDCl_3$

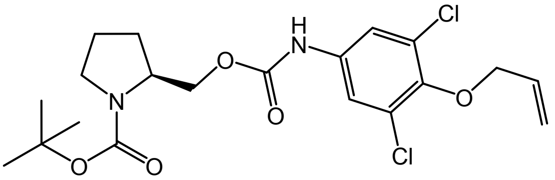

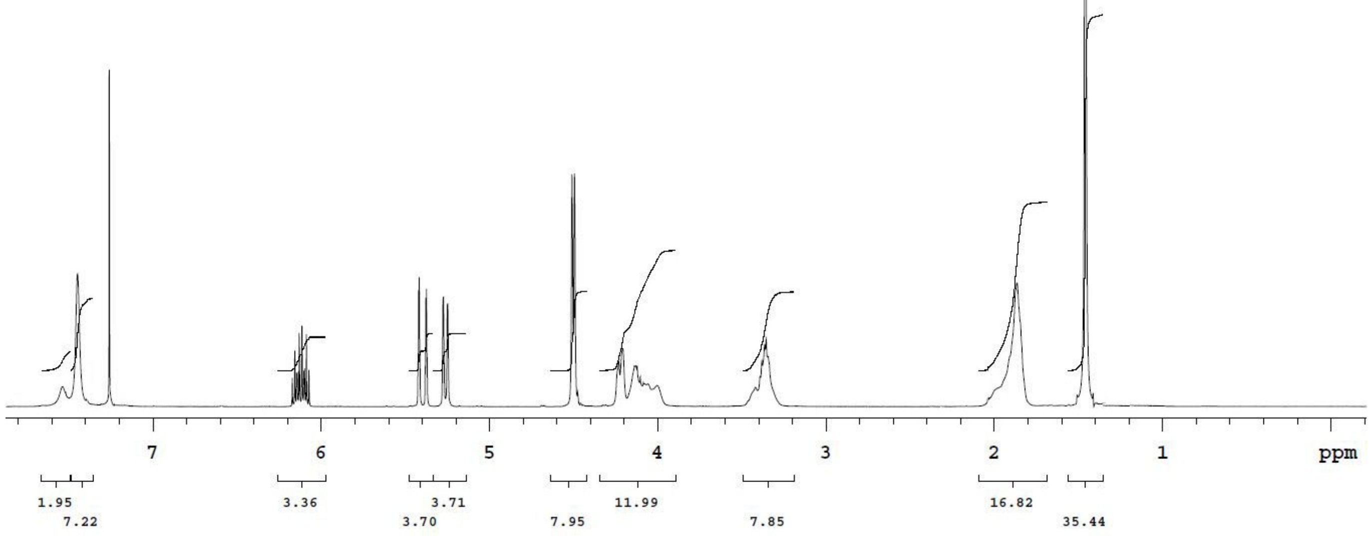

^{13}C (APT) NMR; $CDCl_3$

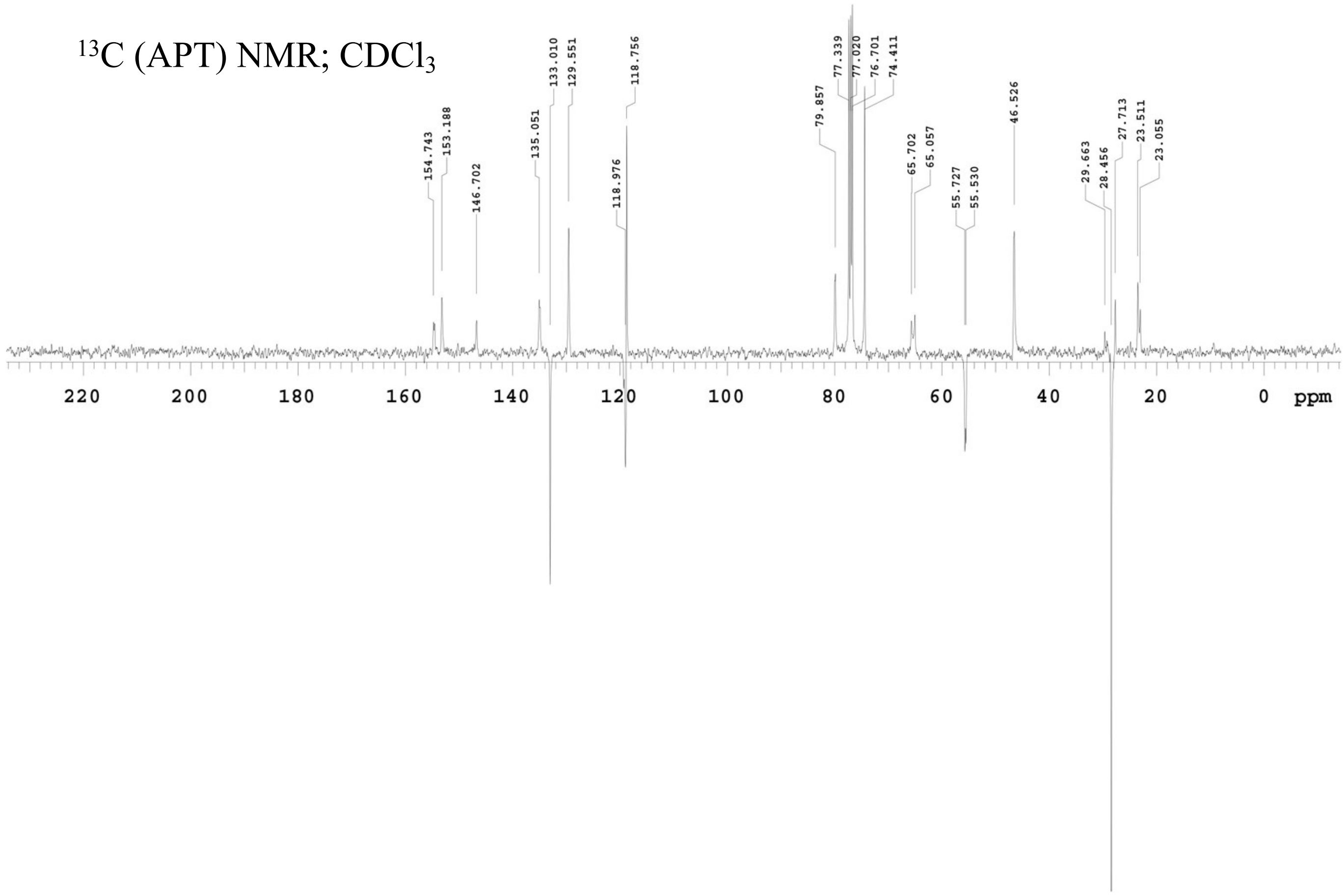

^{1}H NMR; DMSO-d_6

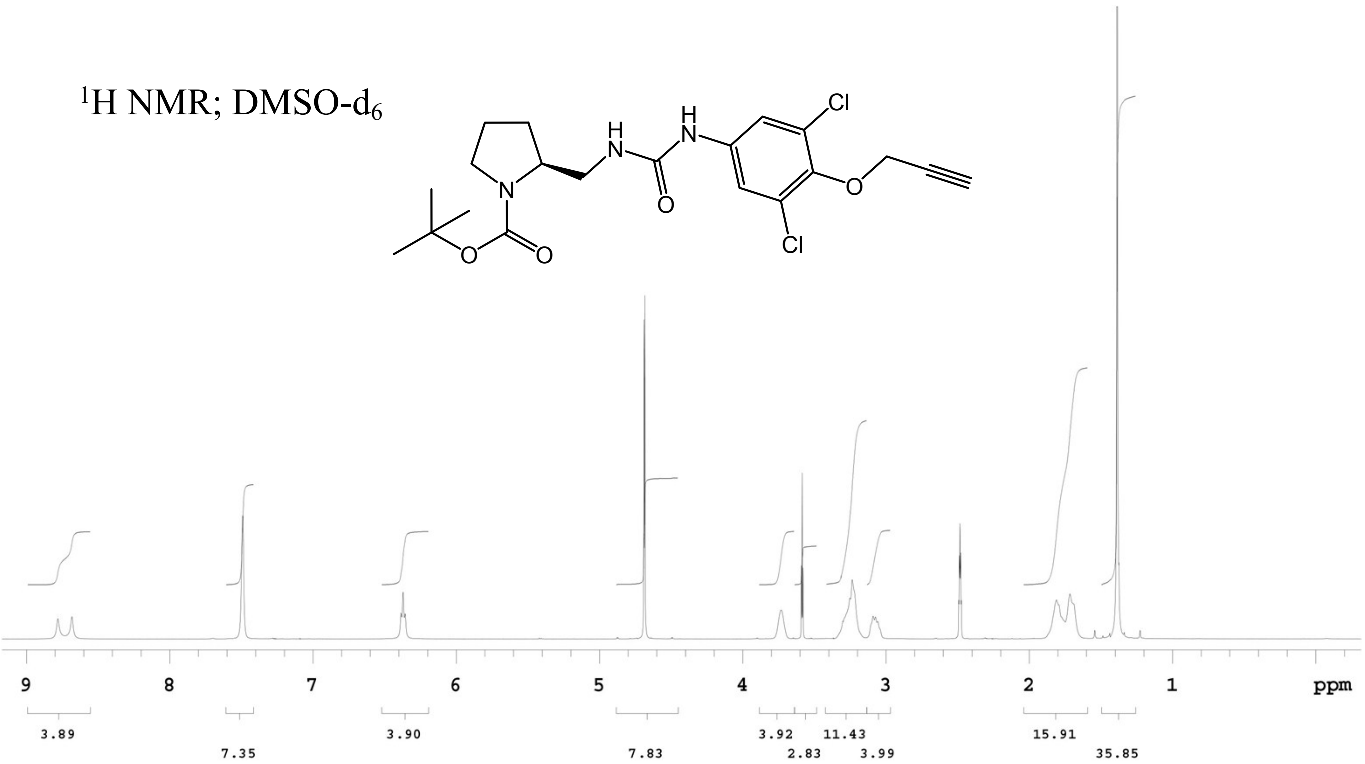

^{13}C NMR; DMSO-d_6

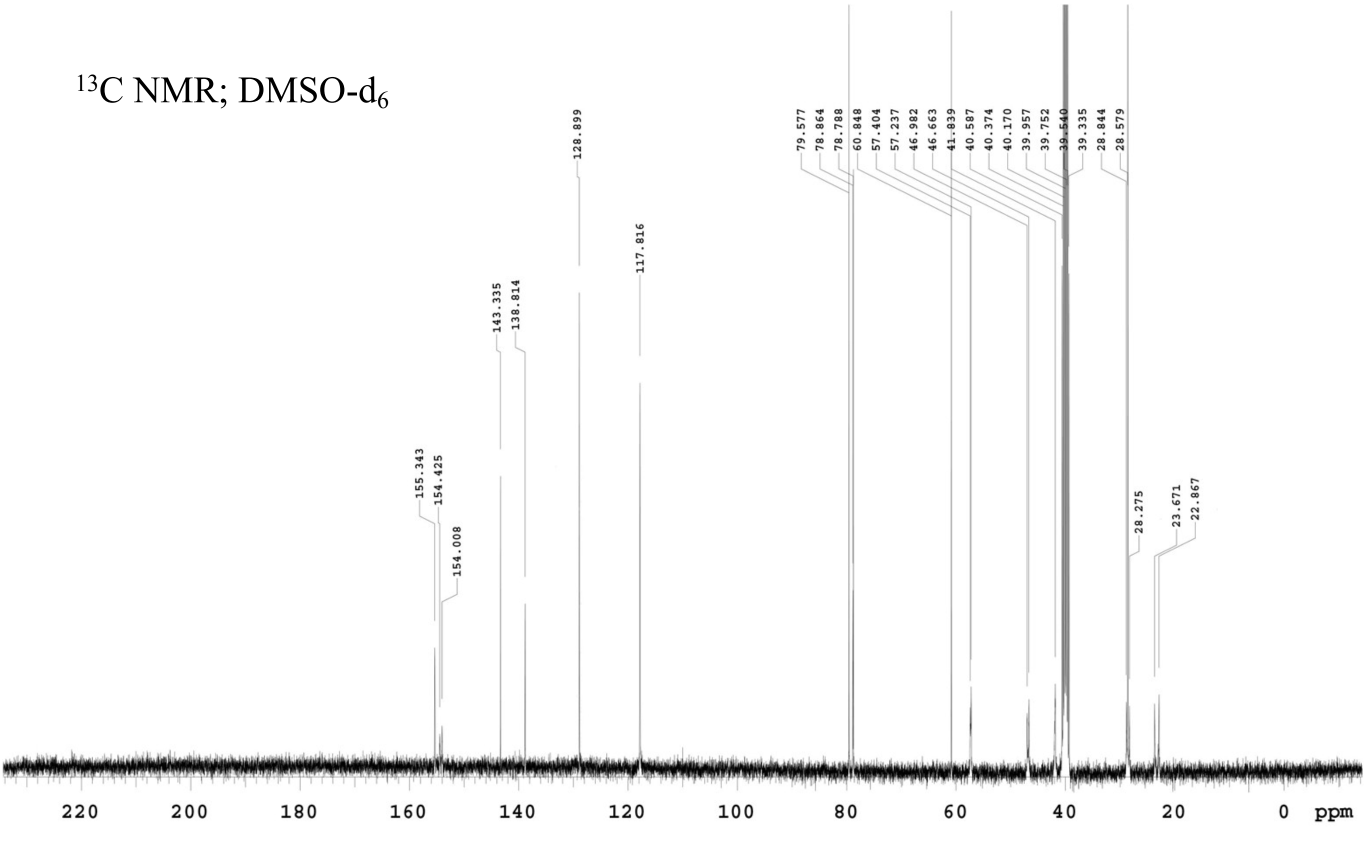

^{1}H NMR; DMSO-d$_6$

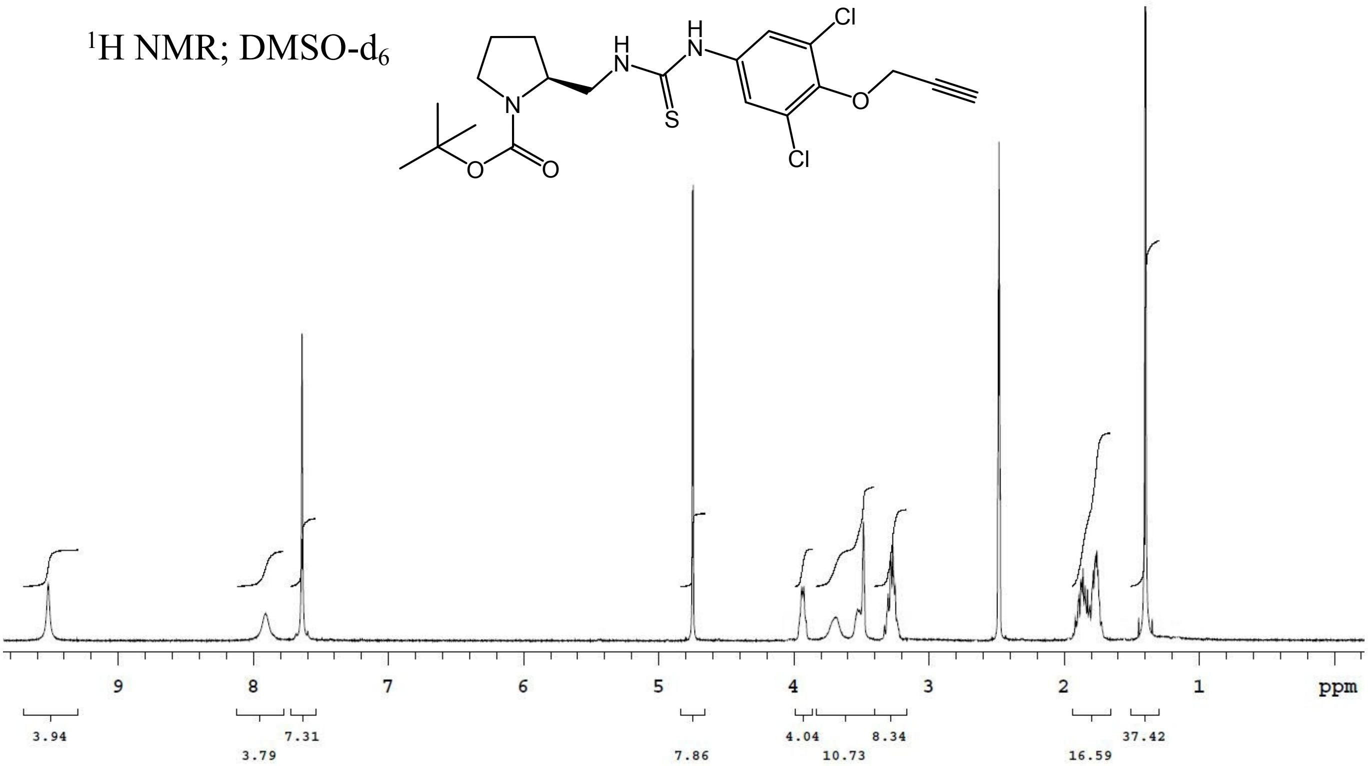

^{13}C NMR; DMSO-d$_6$

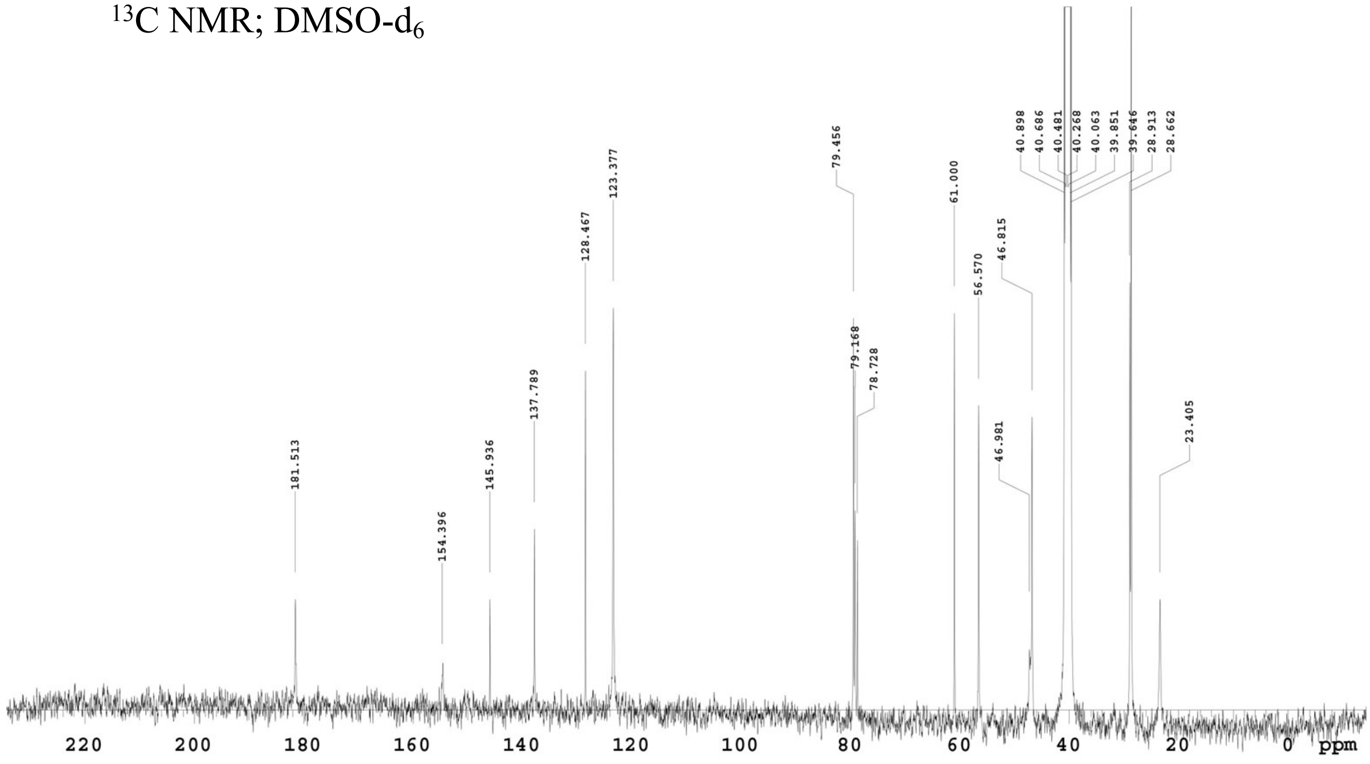

^{1}H NMR; DMSO-d_6

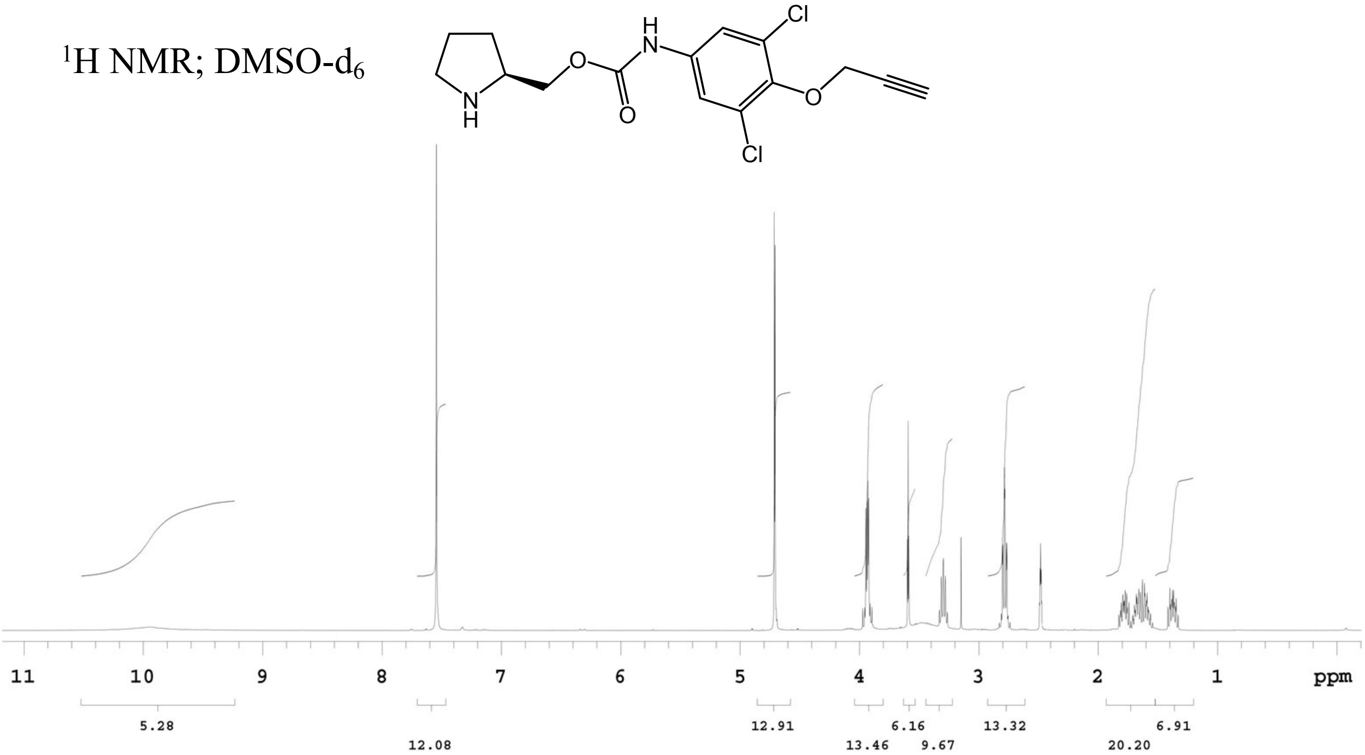

^{13}C NMR; DMSO-d_6

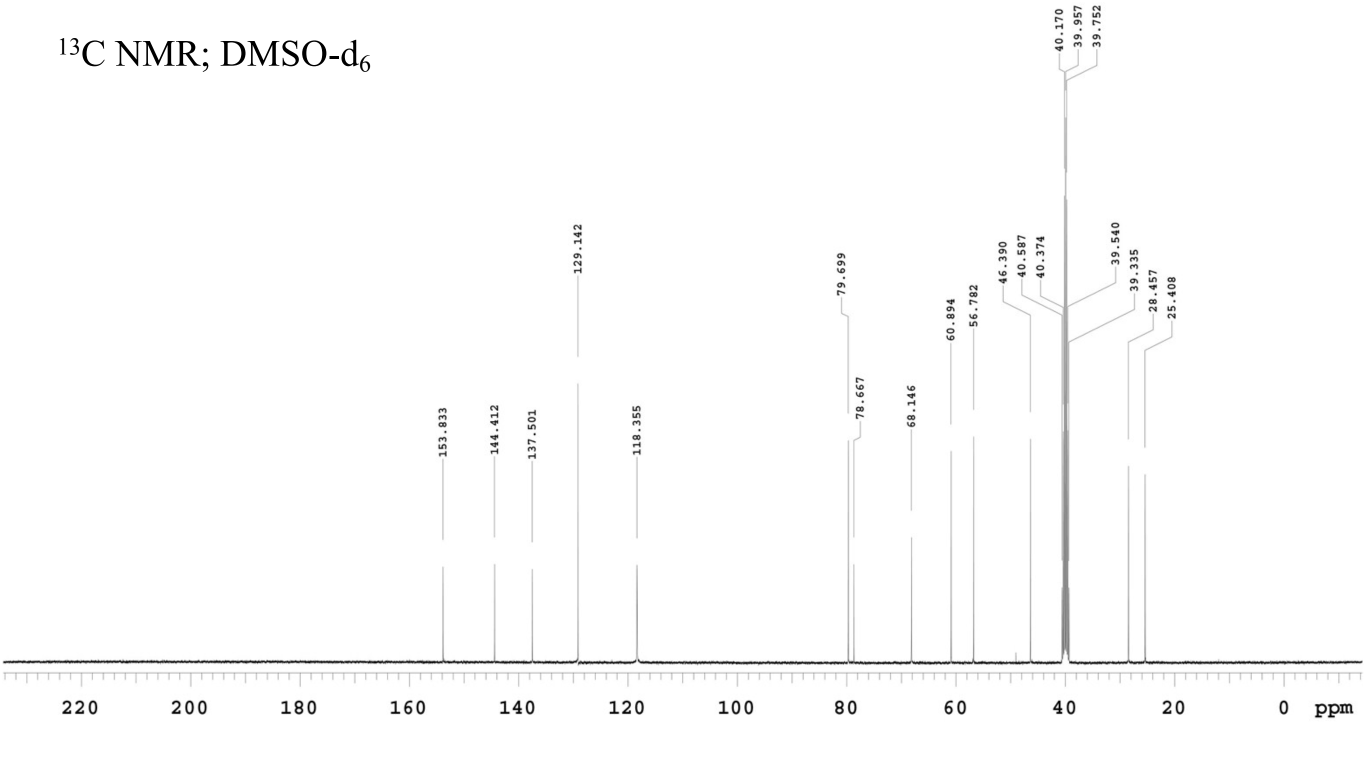

^{1}H NMR; $CDCl_3$

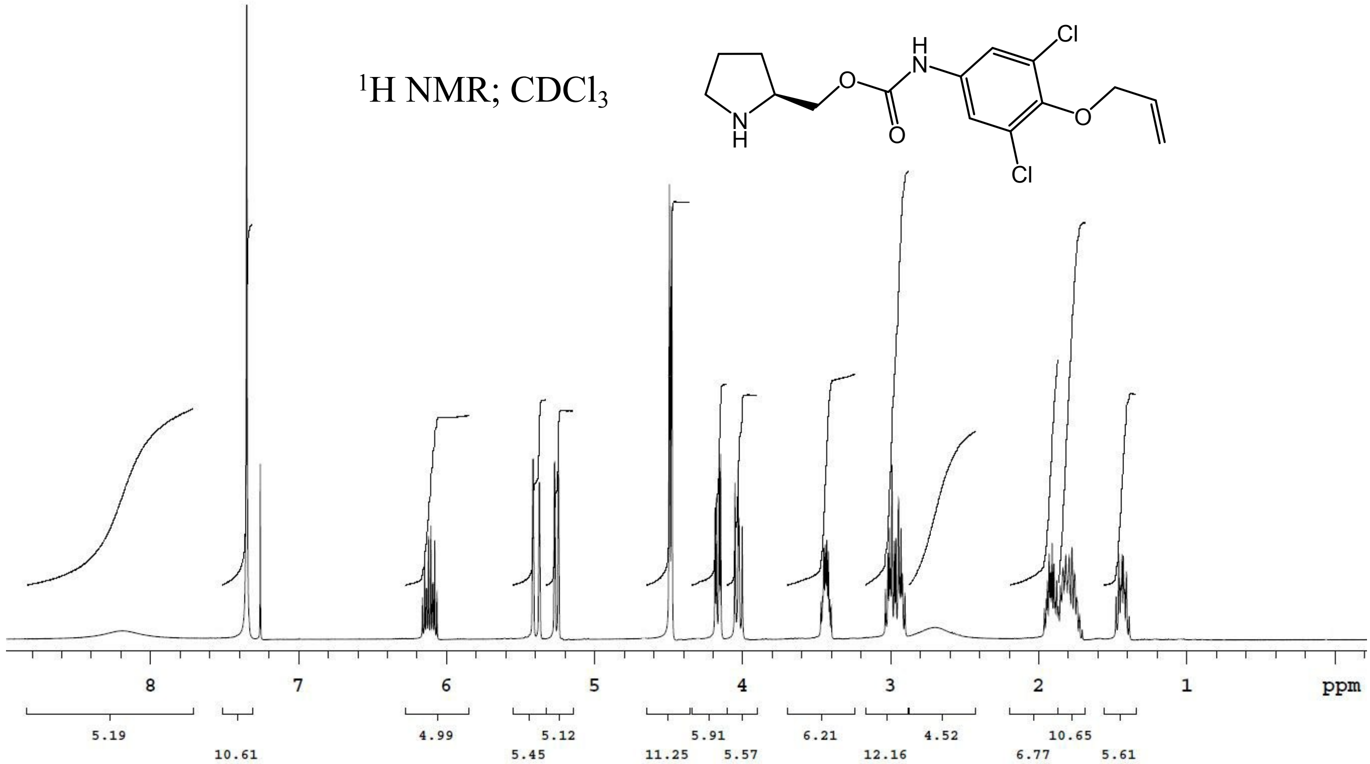

^{13}C (APT) NMR; $CDCl_3$

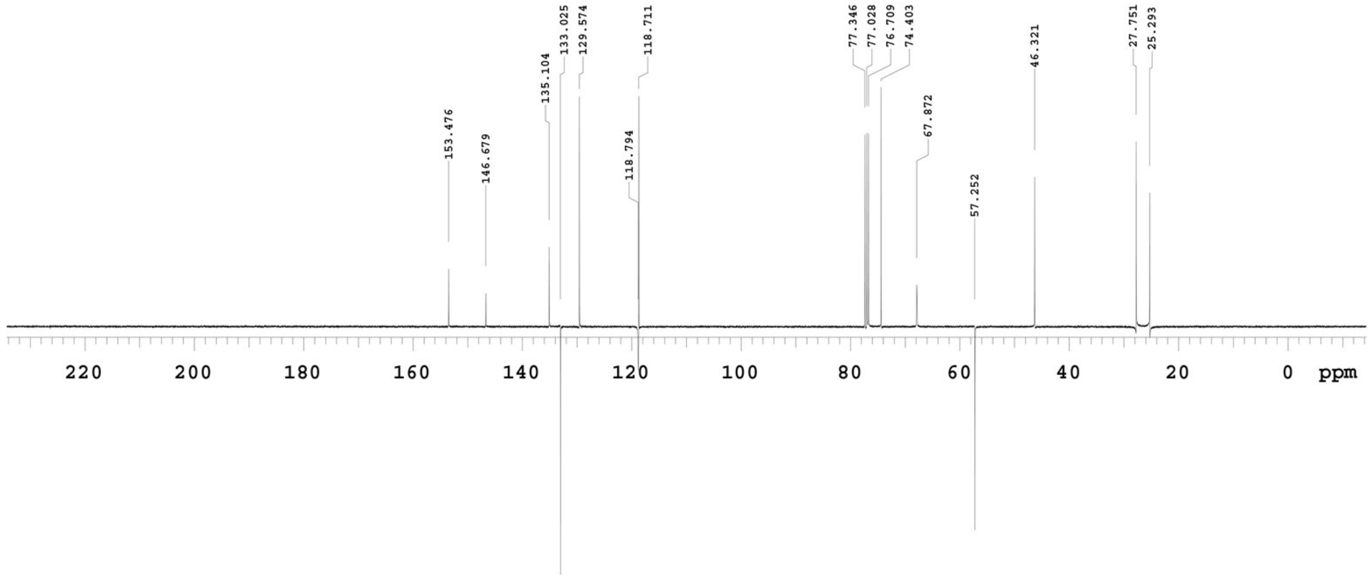

^{1}H NMR; DMSO-d_6

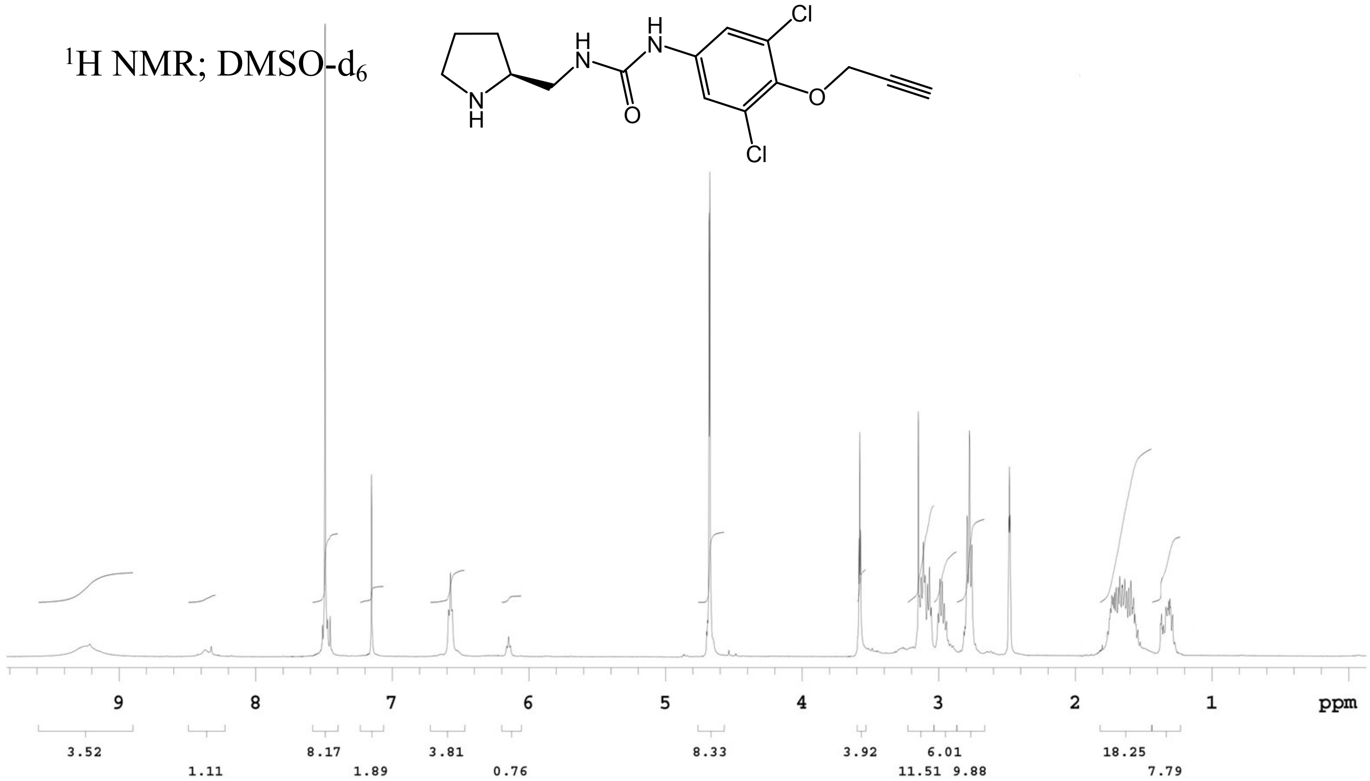

^{13}C NMR; DMSO-d_6

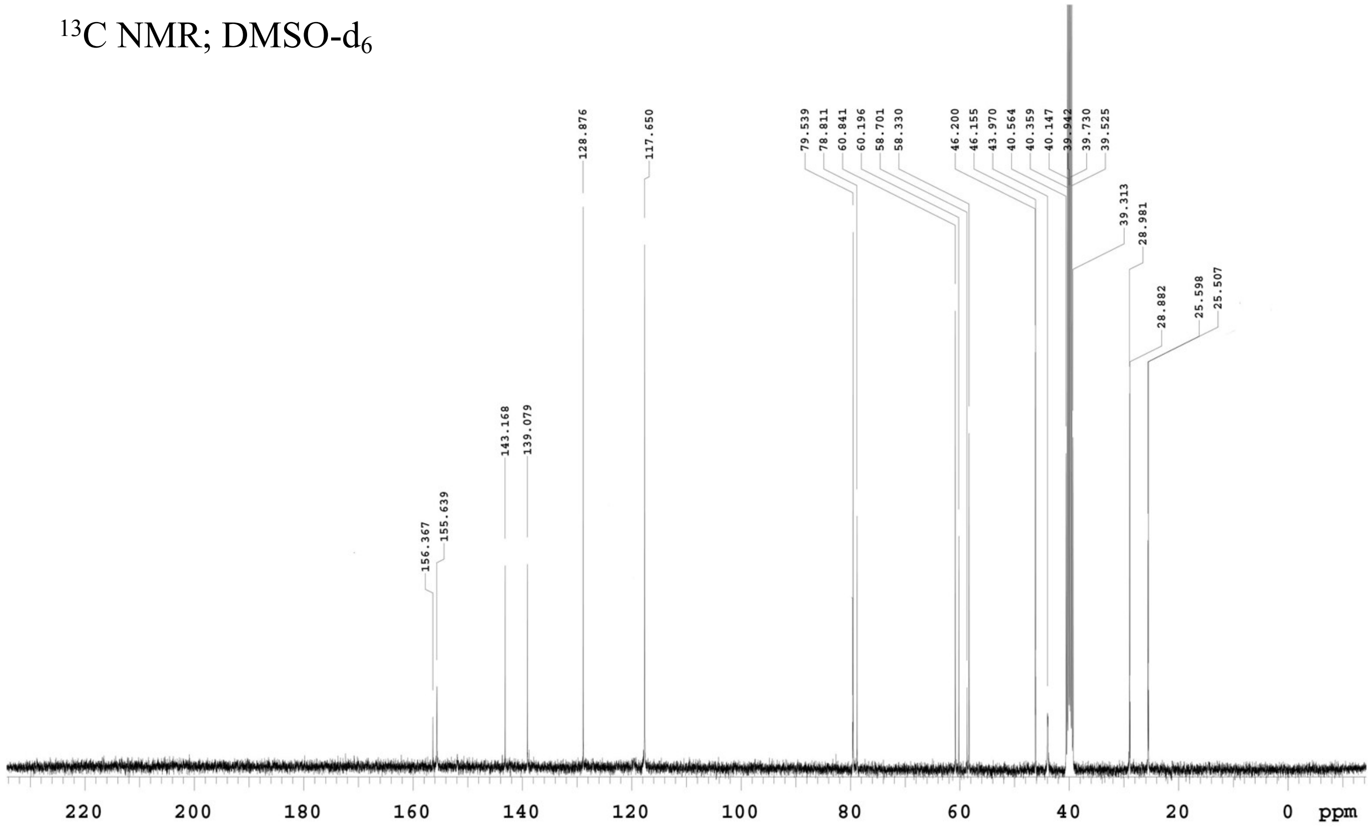

^{1}H NMR; DMSO-d_6

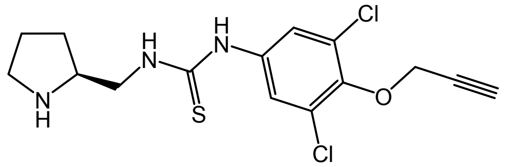

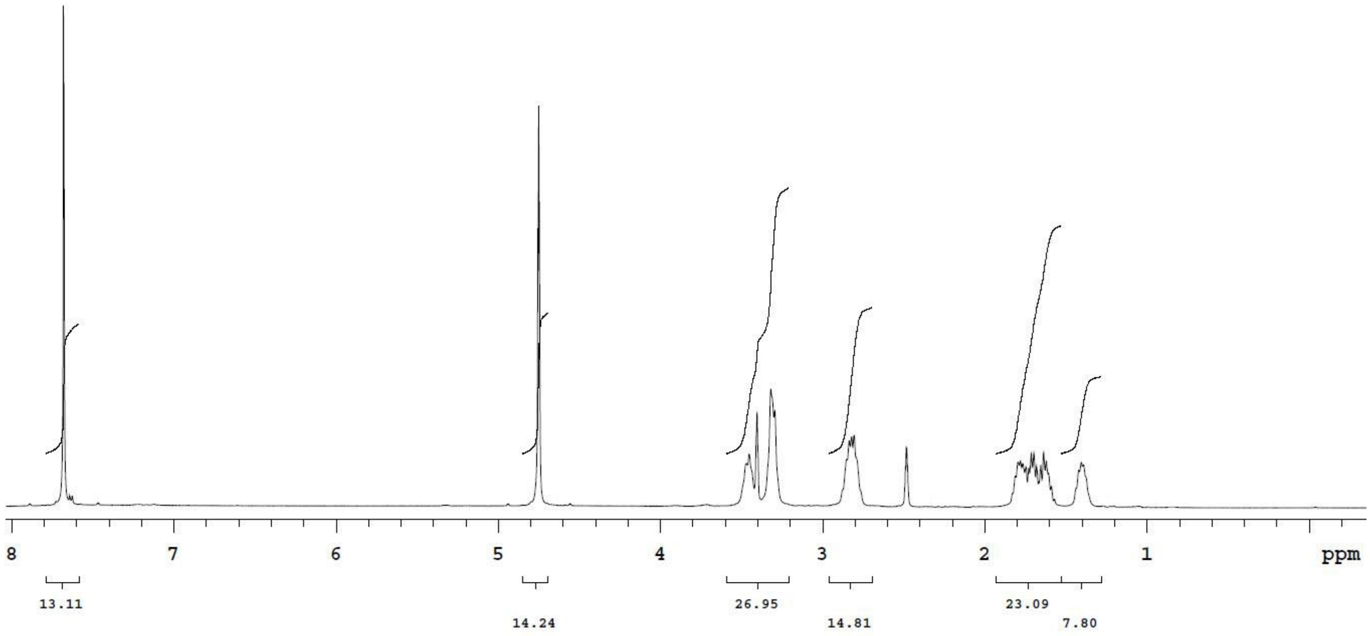

^{13}C NMR; DMSO-d_6

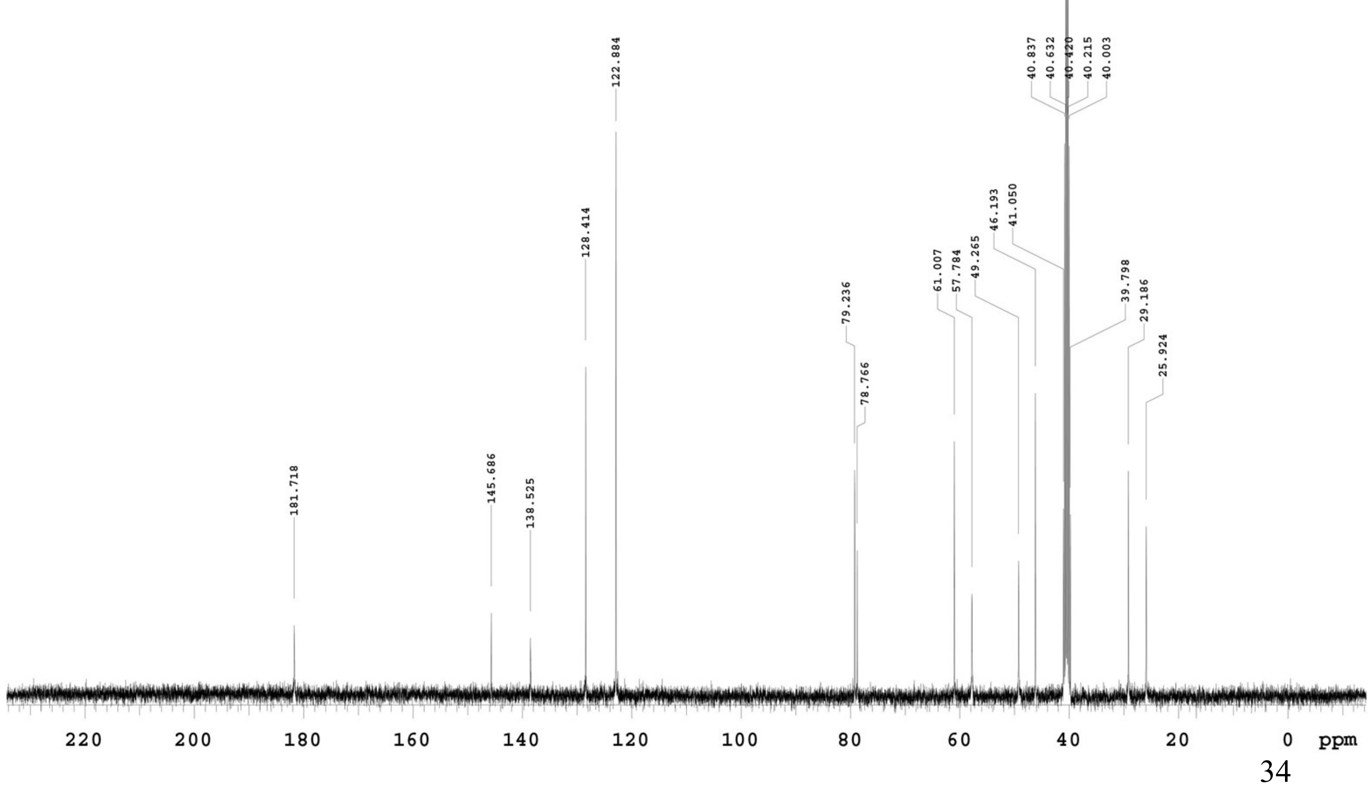

MS spectra

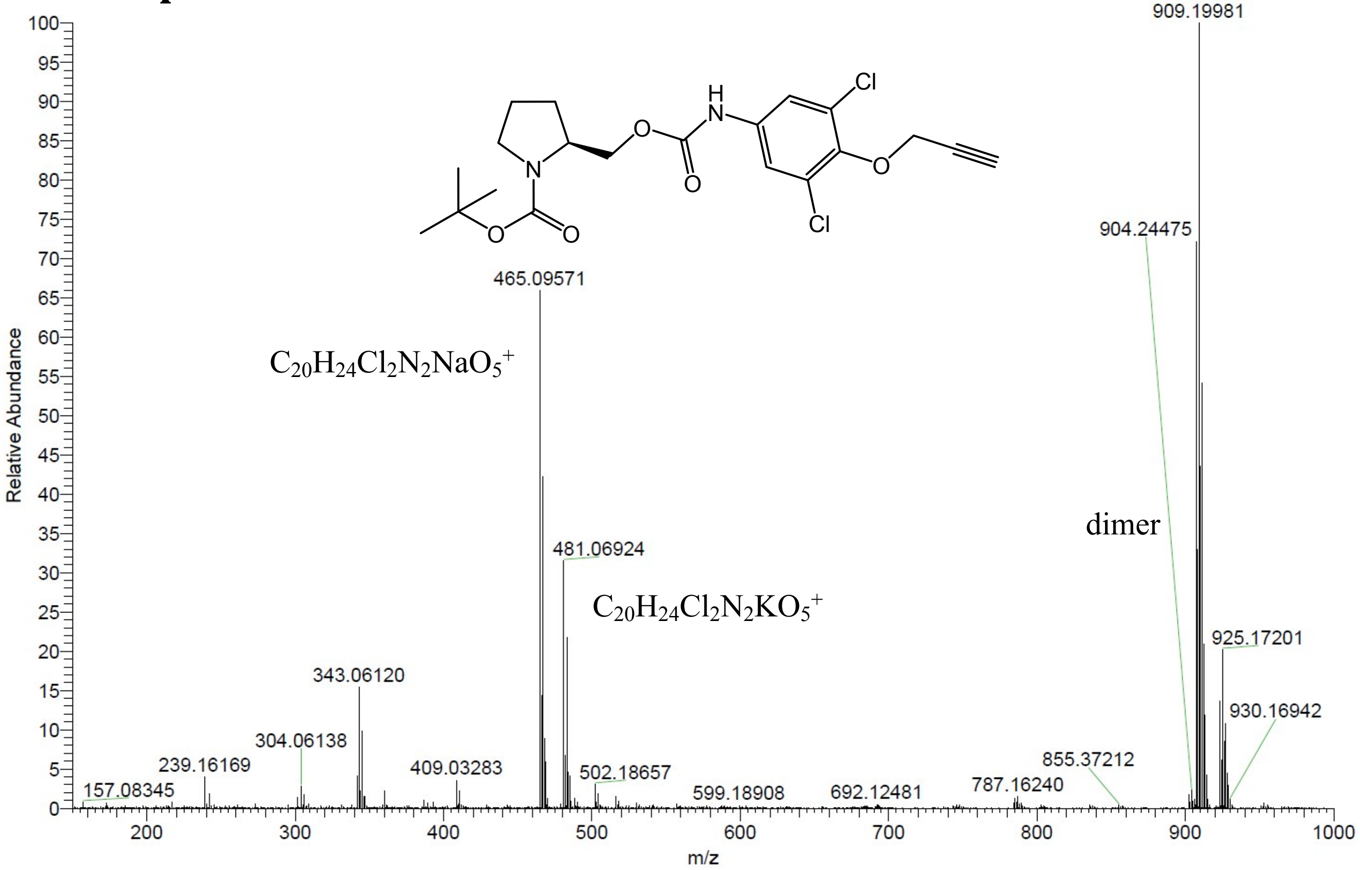

Relative Abundance
m/z
909.19981
904.24475
465.09571
$C_{20}H_{24}Cl_2N_2NaO_5^+$
481.06924
$C_{20}H_{24}Cl_2N_2KO_5^+$
dimer
925.17201
930.16942
343.06120
304.06138
239.16169
409.03283
502.18657
157.08345
599.18908
692.12481
787.16240
855.37212

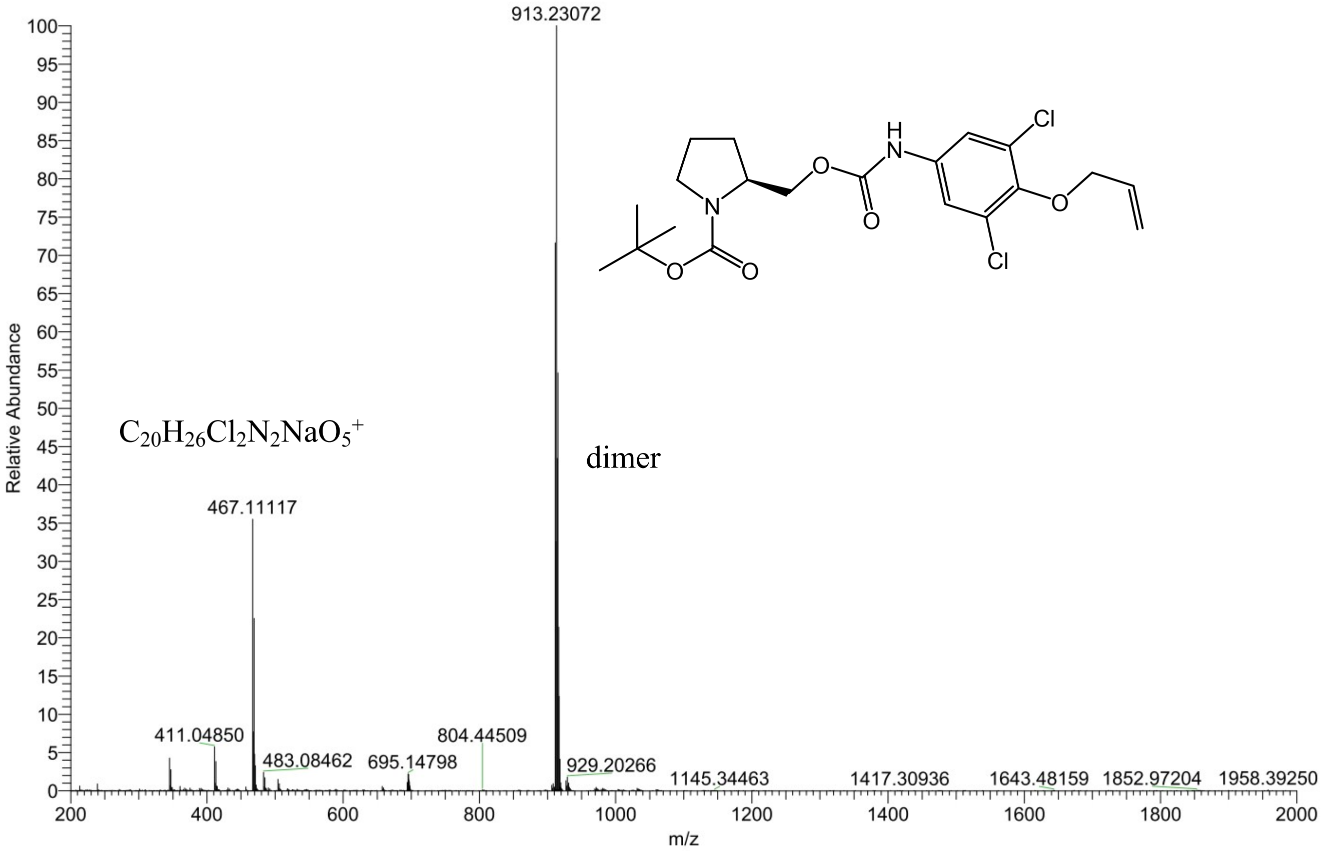

Relative Abundance
m/z
913.23072
$C_{20}H_{26}Cl_2N_2NaO_5^+$
dimer
467.11117
411.04850
483.08462
695.14798
804.44509
929.20266
1145.34463
1417.30936
1643.48159
1852.97204
1958.39250

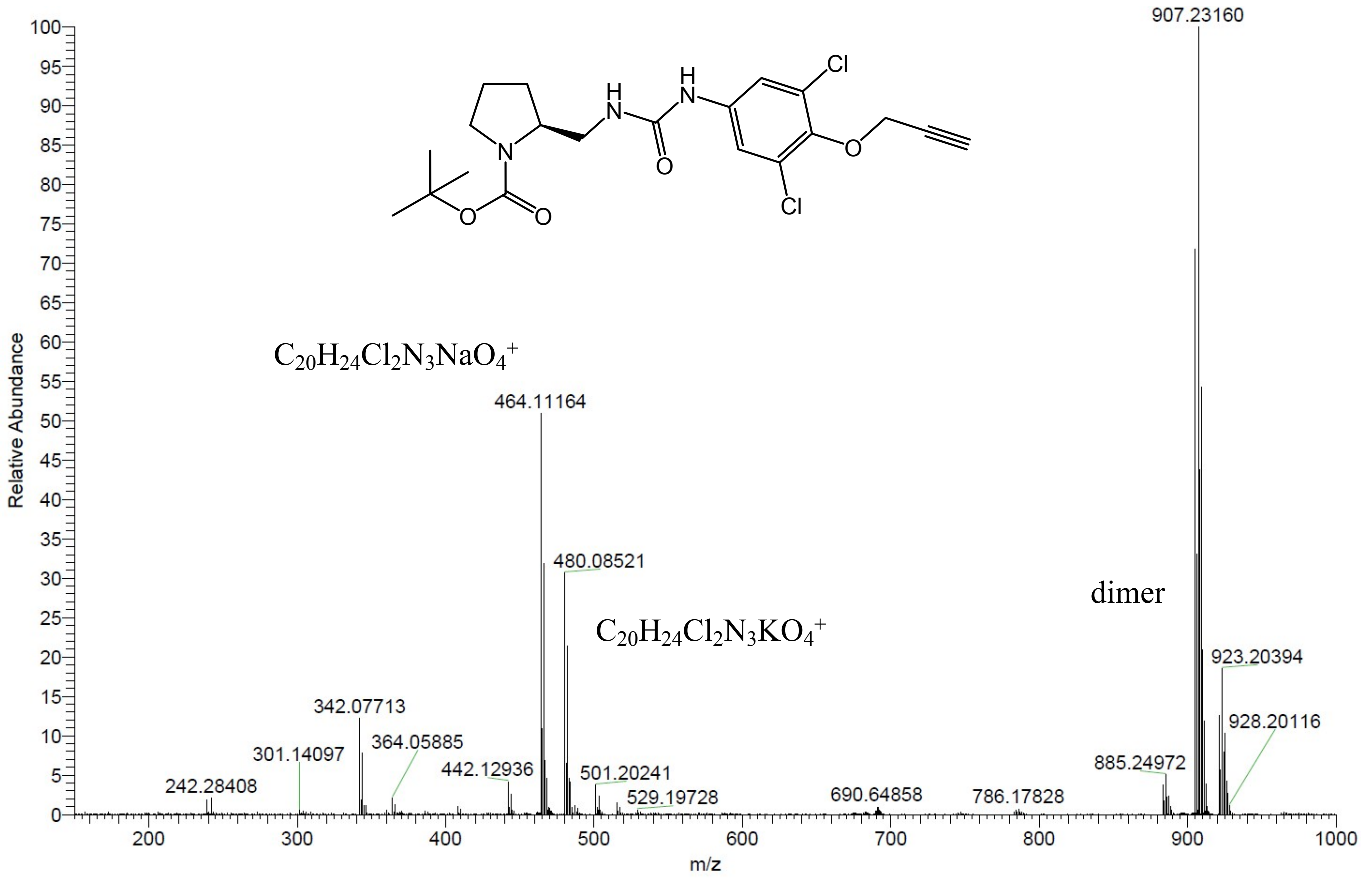

907.23160
C20H24Cl2N3NaO4+
464.11164
480.08521
C20H24Cl2N3KO4+
dimer
923.20394
928.20116
885.24972
342.07713
301.14097
364.05885
442.12936
501.20241
529.19728
242.28408
690.64858
786.17828
Relative Abundance
m/z

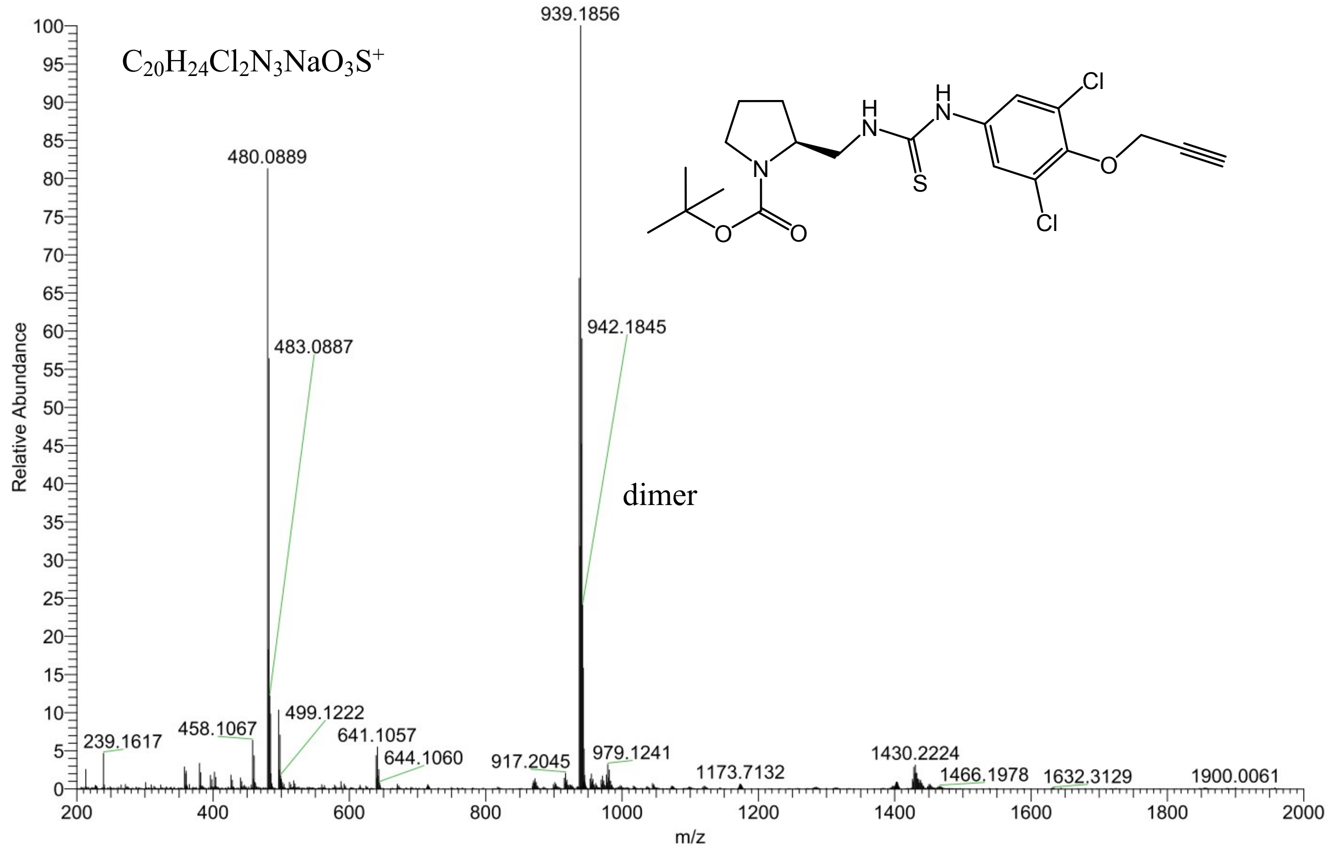

939.1856
C20H24Cl2N3NaO3S+
480.0889
483.0887
942.1845
dimer
499.1222
458.1067
641.1057
239.1617
644.1060
917.2045
979.1241
1173.7132
1430.2224
1466.1978
1632.3129
1900.0061
Relative Abundance
m/z

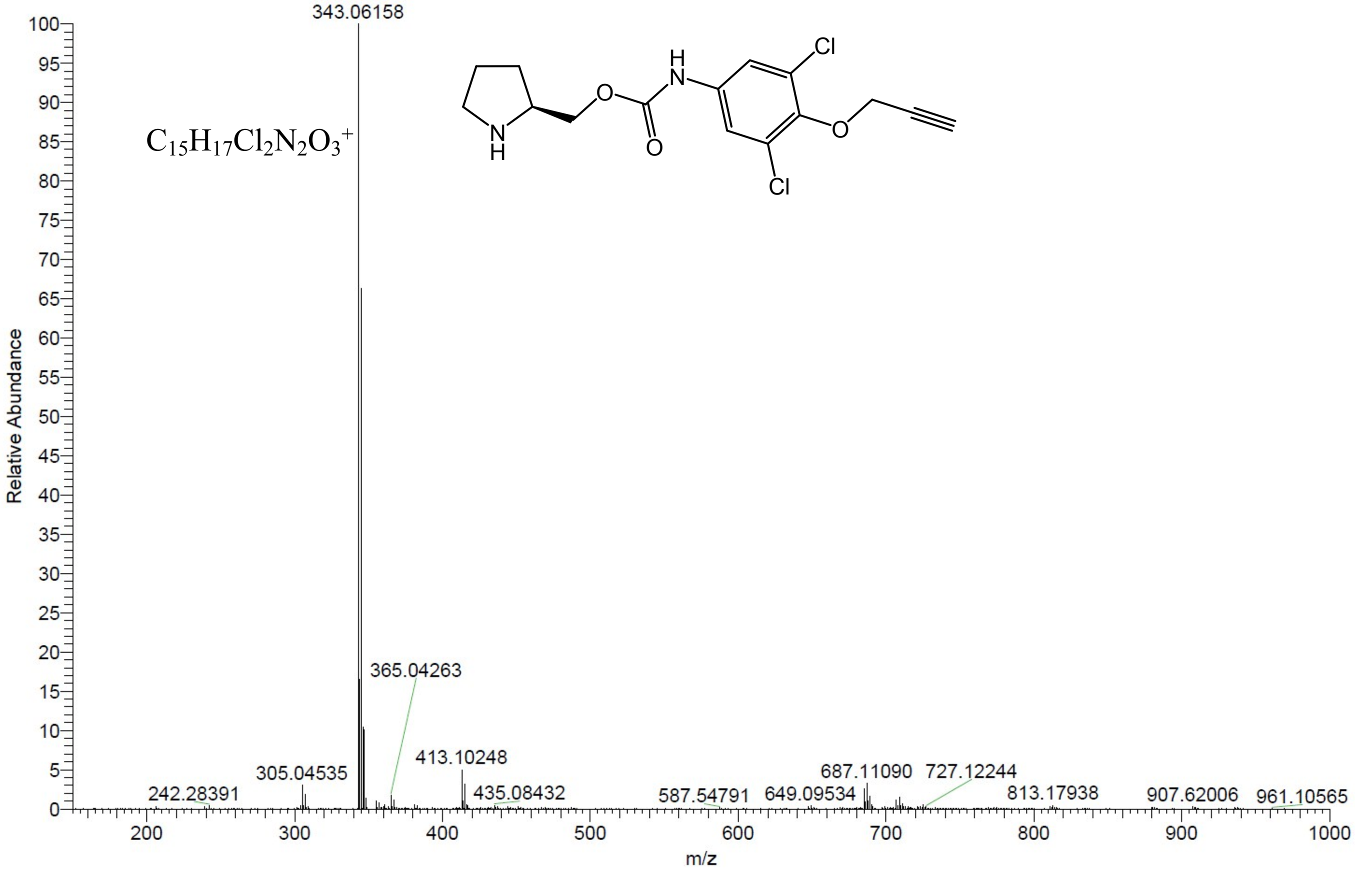
343.06158
$C_{15}H_{17}Cl_2N_2O_3^+$
Relative Abundance
m/z
365.04263
413.10248
305.04535
242.28391
435.08432
587.54791
649.09534
687.11090
727.12244
813.17938
907.62006
961.10565
H
N
O
Cl

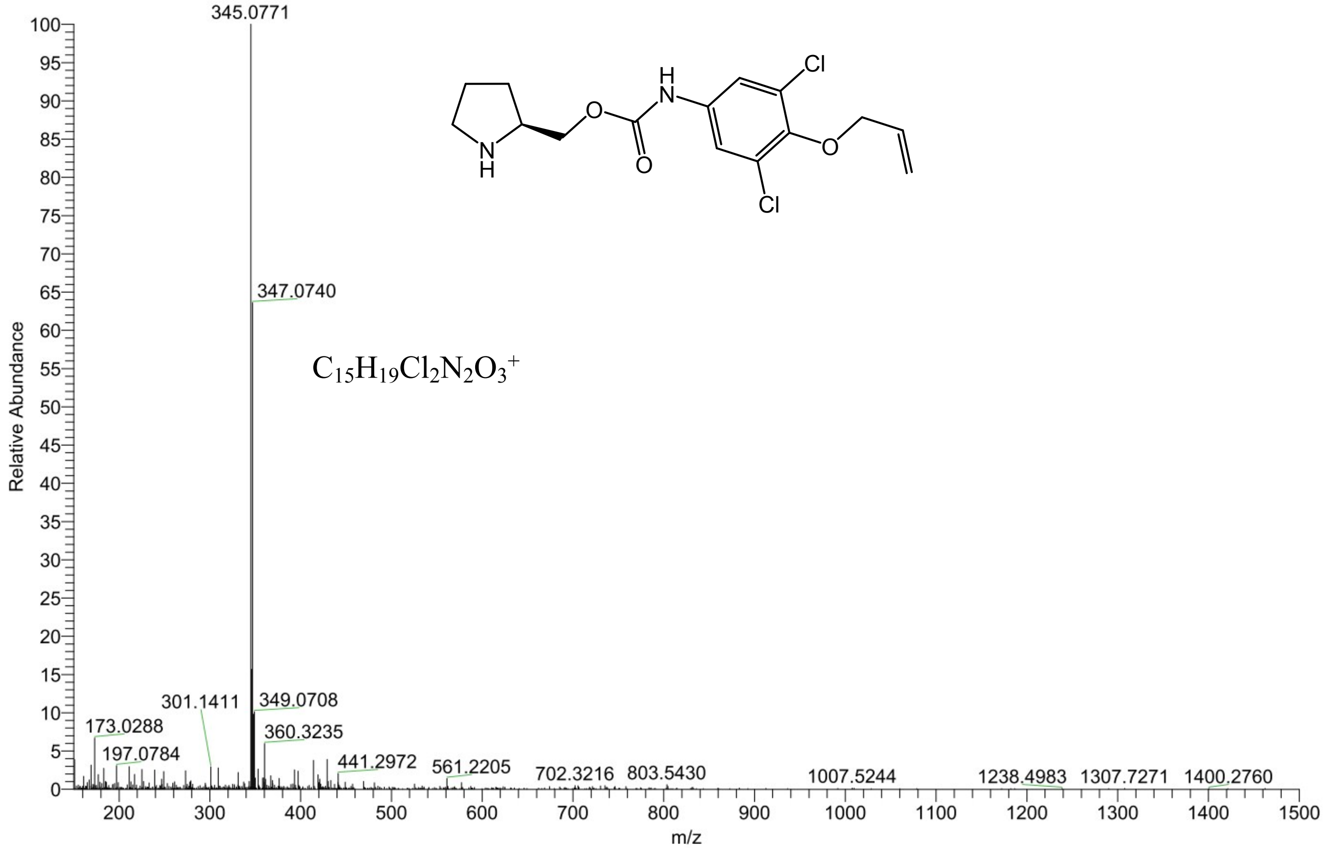
345.0771
347.0740
$C_{15}H_{19}Cl_2N_2O_3^+$
Relative Abundance
m/z
301.1411
349.0708
173.0288
360.3235
197.0784
441.2972
561.2205
702.3216
803.5430
1007.5244
1238.4983
1307.7271
1400.2760
H
N
O
Cl

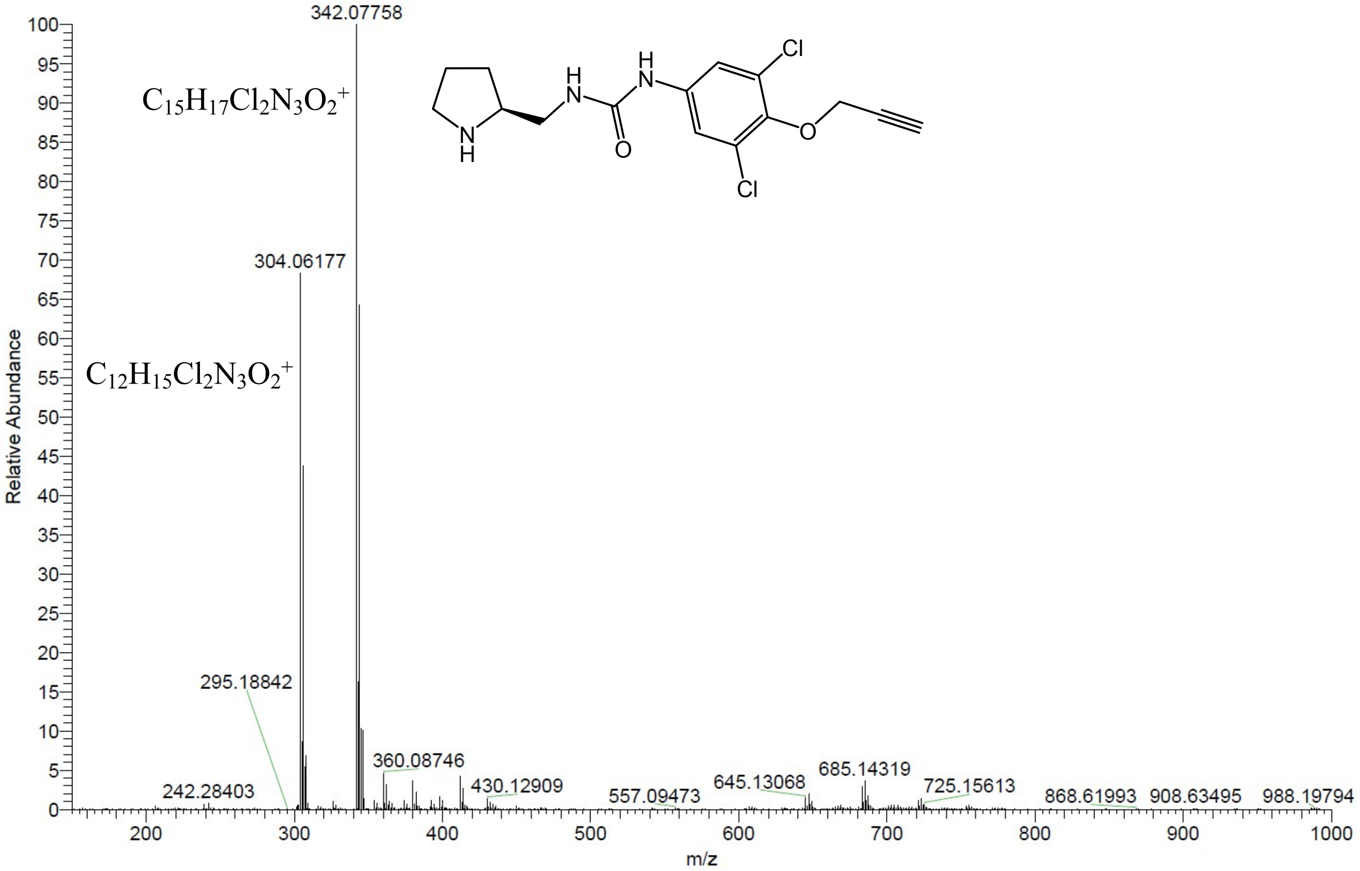
342.07758
C15H17Cl2N3O2+
304.06177
C12H15Cl2N3O2+
295.18842
242.28403
360.08746
430.12909
557.09473
645.13068
685.14319
725.15613
868.61993
908.63495
988.19794
Relative Abundance
m/z
Cl
O
H
N

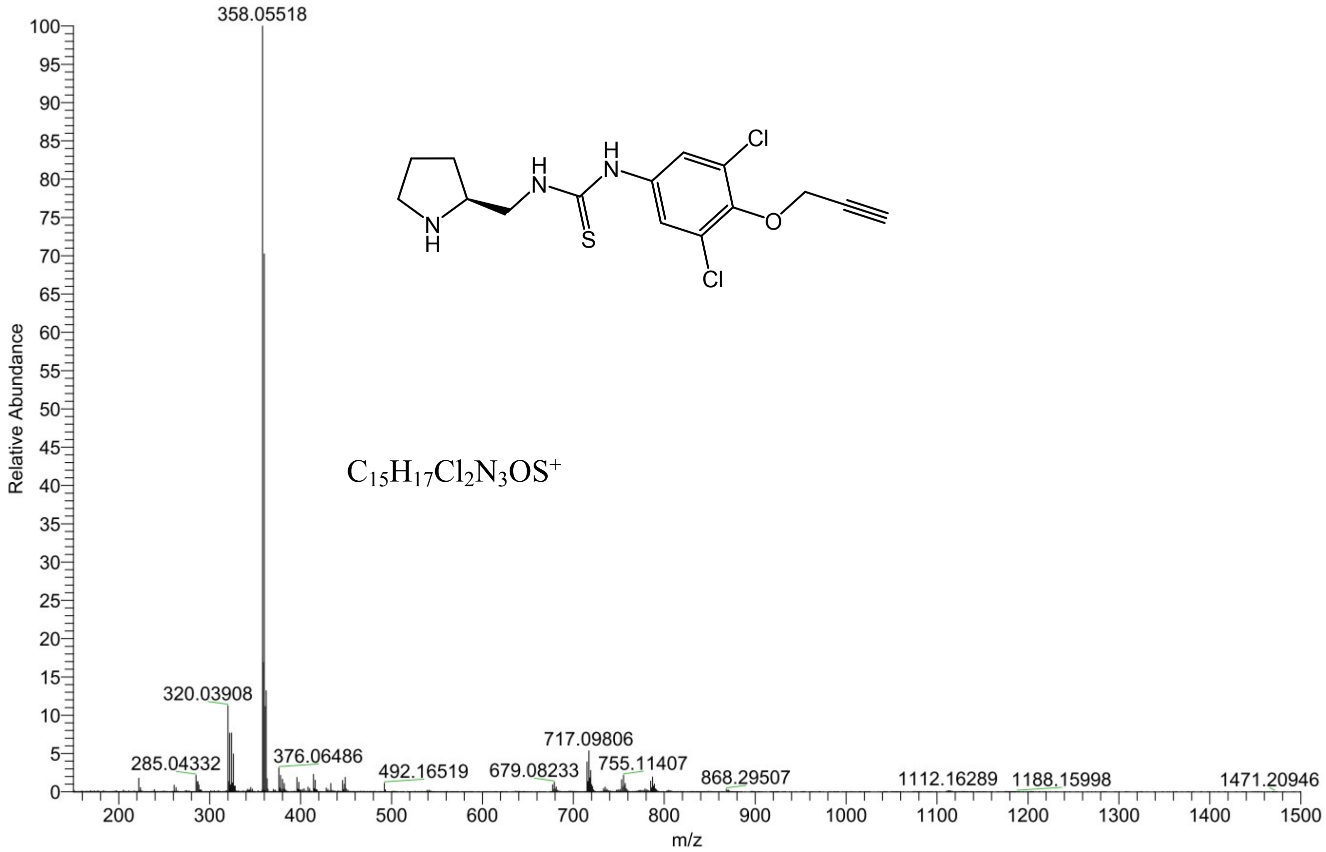
358.05518
C15H17Cl2N3OS+
320.03908
285.04332
376.06486
492.16519
679.08233
717.09806
755.11407
868.29507
1112.16289
1188.15998
1471.20946
Relative Abundance
m/z
Cl
O
S
H
N

References

S1. D. Wolrab, P. Frühauf, M. Kohout and W. Lindner, *J. Sep. Sci.* 2013, **36**, 2826.

S2. H. Munch, J. S. Hansen, M. Pittelkow, J. B. Christensen and U. Boas: *Tetrahedron Lett.* 2008, **49**, 3117.

S3. K. Kacprzak, N. Maier and W. Lindner, *Tetrahedron Lett.* 2006, **47**, 8721.

S4. J. Ivkovic, C. Lembacher-Fadum and R. Breinbauer, *Org. Biomol. Chem.* 2015, **13**, 10456.

S5. D. J. Morriss, A. S. Partridge, C. V. Manville, D. T. Racys, G. Woodward, G. Docherty and M. Wills, *Tetrahedron Lett.* 2010, **51**, 209.

S6. N. Vázquez, V. Gómez-Vallejo, J. Calvo, D. Pardo and J. Llop, *Tetrahedron Lett.* 2011, **52**, 615.

S7. C. Metallinos S. I. Sadraei and N. Zhukovskaya, *Heterocycles* 2014, **88**, 347.
